# DNAJB8 in small extracellular vesicles promotes Oxaliplatin resistance through TP53/MDR1 pathway in colon cancer

**DOI:** 10.1038/s41419-022-04599-x

**Published:** 2022-02-14

**Authors:** Zheng Wang, Yi Li, Rui Mao, Yu Zhang, Jun Wen, Qian Liu, Yanjun Liu, Tongtong Zhang

**Affiliations:** 1 Department of Colorectal Surgery, National Cancer Center/National Clinical Research Center for Cancer/Cancer Hospital, Chinese Academy of Medical Sciences and Peking Union Medical College, Beijing, China; 2 Department of Radiology, The Third People’s Hospital of Chengdu, The Affiliated Hospital of Southwest Jiaotong University, The Second Chengdu Hospital Affiliated to Chongqing Medical University, Chengdu, Sichuan Province China; 3 Department of Dermatology, Xiangya Hospital, Central South University, Changsha, China; 4 State Key Laboratory of Molecular Oncology, National Cancer Center/National Clinical Research Center for Cancer/Cancer Hospital, Chinese Academy of Medical Sciences and Peking Union Medical College, Beijing, China; 5 The Center of Gastrointestinal and Minimally Invasive Surgery, Department of General Surgery, The Third People’s Hospital of Chengdu, The Affiliated Hospital of Southwest Jiaotong University, The Second Chengdu Hospital Affiliated to Chongqing Medical University, Chengdu, China; 6 Medical Research Center, The Third People’s Hospital of Chengdu, The Affiliated Hospital of Southwest Jiaotong University, The Second Chengdu Hospital Affiliated to Chongqing Medical University, Chengdu, Sichuan Province China

**Keywords:** Mechanisms of disease, Prognostic markers, Colon cancer

## Abstract

Chemotherapy is one of the most frequently used therapies for the treatment of colon cancer (COAD). However, Oxaliplatin (L-OHP) resistance is a major obstacle to the effective treatment of COAD. Here, we investigated whether DNAJB8, a heat shock protein 40 (HSP40) family protein, could be used for the prognosis and therapy of L-OHP resistance in COAD. Treatment with small interfering RNA targeting DNAJB8 could restore the response to L-OHP in vitro and in vivo. On the mechanism, we demonstrated that DNAJB8 could interact with TP53 and inhibit the ubiquitination degradation of TP53, leading to MDR1 upregulation which promotes colon cancer L-OHP resistance. We found that small extracellular vesicle (sEV)-mediated transfer of DNAJB8 from L-OHP-resistant COAD cells to sensitive cells contributed to L-OHP resistance. A prognostic signature based on the DNAJB8 levels in both tissue and serum showed that COAD patients with high-risk scores exhibited significantly worse overall survival and disease-free survival than patients with low-risk scores. These results indicate that DNAJB8 levels in serum sEVs may serve as a biomarker for COAD. DNAJB8 from sEVs might be a promising therapeutic target for L-OHP resistance and a prognostic predictor of clinical response.

## Introduction

Colon cancer is one of the most commonly diagnosed cancers and a leading cause of death worldwide [1]. The overall incidence and mortality rates of colon cancer have been declining; however, the prognosis of colon cancer remains poor [2]. Identification of novel effective chemotherapy drugs or improvement of the efficacy of anti-colon cancer drugs would be very helpful for colon cancer treatment, especially for advanced colon cancer.

Oxaliplatin (L-OHP)-based chemotherapy is routinely used to treat colon cancer patients who are at high risk of recurrence or those with advanced or metastatic disease [3]. However, a significant proportion of patients receiving L-OHP therapy become chemoresistant. Therefore, understanding the mechanisms underlying L-OHP resistance can help us to identify a subgroup of patients who may benefit from L-OHP therapy and avoid overtreatment.

Small extracellular vesicles (sEVs) are nanovesicles with a diameter of 40–150 nm. They are released into the extracellular environment via the endosomal vesicle pathway by fusion with the plasma membrane. A broad range of cells secrete sEVs, including T/B, epithelial, dendritic, and tumor cells [4]. sEVs are essential for intercellular communication [5]. Tumor-derived sEVs deliver DNA fragments, RNA, proteins, and lipids, which have been reported to play a major role in cancer progression, including chemoresistance [6]. However, whether sEVs derived from resistant colon cancer cells can confer drug resistance to sensitive cells needs to be elucidated.

DNAJ (heat shock protein 40 [HSP40]) homolog, subfamily B, member 8 (DNAJB8) belongs to the DNAJ/HSP40 family of proteins, which regulates chaperone HSP70 activity [7]. DNAJB8 suppresses aggregation of polyglutamine proteins through its C-terminal tail [8, 9]. Recently, it has been reported that DNAJB8 is a testicular cancer antigen and a cancer stem-like cell antigen involved in renal cell carcinoma [10, 11]. Our previous comparative genomic hybridization data showed that the *DNAJB8* gene is more highly expressed in metastatic colon cancer compared with primary tumors [12].

The clinicopathological significance of DNAJB8 and its association with L-OHP resistance in COAD has not been elucidated. The aim of the present study is to investigate the contributions of DNAJB8 to L-OHP resistance and explore the therapeutic implications for L-OHP-resistant COAD patients.

## Material and methods

### COAD samples and cell lines

216 tissue samples of fresh colon cancer with matched colon mucosa were obtained from the Cancer Hospital, Chinese Academy of Medical Sciences and Peking Union Medical College (CAMS and PUMC) from 2009 to 2012. The mean age of the patients was 60.4 years, comprising 109 males and 107 females. None of the patients with COAD had received preoperative chemotherapy and/or radiotherapy. After the operation, the tumors were histologically classified and staged according to the tumor node metastasis (TNM) system, and the histological grade of tumors was defined according to the tumor differentiation. Blood samples were collected from patients to isolate extracellular vesicles from serum. Blood specimens were immediately processed by centrifugation at 2,000 × *g* for 20 min at room temperature and serum was separated and frozen in aliquots at −80 °C. Informed consent was obtained from all of the participants. This study was approved by the Ethics Committee/Institutional Review Board of the Cancer Institute/Hospital, Peking Union Medical College and Chinese Academy of Medical Sciences (approval no. NCC2017G-023). And all participants provided written informed consent prior to their enrollment in the study.

COAD cell lines, SW480 and SW620 were purchased from the Institute of Cell Biology, Chinese Academy of Sciences (Shanghai, China) and maintained in RPMI1640 medium supplemented with 10% fetal bovine serum (FBS) at 37 °C in a 5% CO_2_ incubator.

### Development of L-OHP-resistant colon cancer cell lines

L-OHP (Sanofi-Synthelabo, Hangzhou, China) was purchased from the pharmacy at the Chinese Academy of Medical Sciences. SW480 and SW620 cells were exposed to an initial L-OHP concentration of 5 μg/L in RPMI1640 plus 10% FBS. After a 24­h incubation, the old media was discarded and fresh media was added. The surviving population of cells was grown to 80% confluence and passaged to ensure viability. The concentration of L-OHP that the surviving population was exposed to was then sequentially increased in the same manner to 10 μg/L (15days), 20 μg/L (30 days), and finally to the clinically relevant serum concentration of 100 μg/L. For all of the in vitro and in vivo studies, SW480 and SW620 L­OHP-resistant cells were used at no higher than 5 passages from creation and were maintained and exposed to 100 μg/L L-OHP unless otherwise indicated.

### Quantitative real-time PCR (qRT-PCR)

Total RNA was reverse-transcribed into cDNA with random primers using the Transcriptor First Strand cDNA Synthesis Kit (Roche, Penzberg, Germany) according to the manufacturer’s instructions. mRNA expression was quantified via qRT-PCR using FastStart Essential DNA Green Master Mix (Roche, Penzberg, Germany) on a Roche LightCycler 480 (Roche, Penzberg, Germany). Relative levels were determined using the 2^−ΔΔCt^ method, and the mRNA levels were normalized to the GAPDH levels. Divergent primers, rather than the more commonly used convergent primers, were designed to broadly target the mRNAs (Table S1). Primer specificity was verified using BLAST, with a single peak in the melting curve indicating the generation of a specific product. Three experimental replicates were performed for each sample. Relative expression was determined using inter-experiment normalization to GAPDH.

### siRNA transfection

siRNA oligonucleotide duplexes and scramble control siRNA were synthesized by GenePharma, Suzhou Co, Ltd (Suzhou, P.R. China). COAD cells at 70% confluence were transfected with the indicated siRNA with Lipofectamine RNAiMAX (Thermo Fisher Scientific, Waltham, MA, USA). The siRNA sequence targeting at DNAJB8 was 5′-TGGAAAATCCCTCAGATTTCTCC-3′, and the siRNA sequence targeting at TP53 was 5′-CCCAAGCAATGGATGATTTGATG-3′.

### Purification and quantification of sEVs

sEVs were purified from serum and cell lines by differential centrifugation as previously described [13]. Briefly, culture medium (CM) was subjected to sequential centrifugation steps at 800 × *g* and 2000 × *g*. The resulting supernatant was filtered using a 0.2-μm filter and concentrated 20 times using a Vivaflow 200 R crossflow unit (Sartorius) with a 100,000 kDa cutoff filter. Then, the serum and CM were centrifuged at 16,500 × *g* for 30 min. Ultracentrifugation was performed at 100,000 ×*g* for 16 h at 4 °C. The supernatant was removed and PBS was added to the pellet for an overnight washing step. The resultant sEV pellet was resuspended in PBS and harvested for subsequent analyses.

In addition, sEVs were purified using an OptiPrep™ density gradient. Briefly, a discontinued iodixanol gradient was set by diluting a stock of OptiPrep™ (60% w/v) with 0.25 M sucrose/10 mM Tris, pH 7.5 to generate 40%, 20%, 10%, and 5% w/v iodixanol solutions. The gradient was layered using 3-mL fractions each of 40%, 20%, 10%, and 5% w/v iodixanol solution. sEVs obtained after differential centrifugation were overlaid on the top of 5% w/v iodixanol solution and spun at 100,000 × *g* at 4 °C for 18 h. Fractions of 1 mL were collected from the top of the tube, diluted with 1.5 mL of 1× PBS, and further subjected to centrifugation at 100,000 × *g* at 4 °C for 1 h. The pellet obtained was again washed with 1 mL 1× PBS and centrifuged at 100,000 × *g* at 4 °C for 1 h to collect sEVs. The control OptiPrep™ gradient was run in parallel to determine the density of each fraction using 0.25 M sucrose/10 mM Tris, pH 7.5. The size distribution and concentration of sEVs were analyzed by nanoparticle-tracking analysis using a ZetaView particle tracker from Particle Metrix (Germany).

### Enzyme-linked immunosorbent assay (ELISA)

The concentration of DNAJB8 from serum was quantified using a DNAJB8 ELISA kit (MyBioSource, San Diego, CA, US) according to the manufacturer’s instructions as described previously. For analysis of DNAJB8 from sEVs, sEVs were first pre-treated with Proteinase K. Equal numbers of sEVs used for protein extraction were suspended in SDS lysis buffer. Then the concentration of DNAJB8 was determined using the ELISA kit. All of the experiments were performed in triplicate.

### Proliferation assay

The cell proliferation assay was performed using the CCK-8 assay according to the manufacturer’s instructions (Sigma-Aldrich, St. Louis, MO, USA). Briefly, SW480 and SW620 cells growing in the logarithmic phase were collected and seeded into 96-well plates at a concentration of 1 × 10^3^ cells per well. After cell attachment, varying concentrations of oxaliplatin were added, and the cells were cultured for an additional 48 h. Then, CCK‐8 (10 μl) was added to the wells, followed by incubation for 2 h. The optical density value at 450 nm was recorded under a microplate reader (Bio‐Rad) from day 1 to day 5.

### Mammosphere formation assay

Mammosphere formation assay have been performed as previously described [14]. Briefly, the ability of cells in the non-adherent population of monolayer cultures to initiate mammosphere formation after L-OHP exposure was assessed by harvesting, washing and resuspending non-adherent cells in phenol red–free DMEM–F12 medium (supplemented with 20 ng/mL bFGF, 10 ng/mL EGF, B27 supplement and N2 supplement). Cells were then passed through a 40-μm sieve, counted, diluted and plated into 96-well plates at clonal densities. Mammospheres were counted on day 5.

### Flow cytometry

SW480 and SW620 cells growing in the logarithmic phase were collected and seeded into 6-well plates. After cell attachment, 1000 µg/L of oxaliplatin was added into the SW480 cells and 300 µg/L of oxaliplatin into the SW620 cells for an additional 48-h culture. Apoptosis rate was analyzed by flow cytometry using an Annexin V‐fluorescein isothiocyanate (FITC)/propidium iodide (PI) apoptosis detection kit (BD Biosciences). In short, SW480 and SW620 cells were collected and then re‐suspended in 1× binding buffer (500 μl) containing Annexin V‐FITC (20 μl) and PI (10 μl) in the darkness for 15 min at room temperature. Subsequently, apoptotic cells were evaluated by a fluorescence-activated cell sorting analyzer (Becton Dickinson). The results were analyzed using FlowJo 7.6.1 software.

### Cell fractionation

2 × 10^6^ cells were lysed in a hypotonic buffer (10 mM HEPES (pH 7.9), 10 mM KCl, 1.5 mM MgCl_2_, 0.5 mM DTT). After centrifugation, the cytosolic proteins in the supernatant were collected and the nuclear pellets were extracted with a high salt buffer (20 mM HEPES (pH 7.9), 20% glycerol, 0.2 mM EDTA, 300 mM KCl, 1.5 mM MgCl_2_, 0.5 mM DTT) for 30 min on ice, followed by centrifugation to collect the nuclear extracts. The proteins of the cytoplasmic fraction and nuclear fraction were subjected to western blot analysis.

### Western blot analysis

Total cell lysates were isolated using RIPA Reagent following the manufacturer’s instructions, clarified (12,000 g, 30 min, 4 °C), denatured, and subjected to SDS-PAGE and western blot analysis. Antibodies used were anti-P53 (Cell Signaling Technology, 2524 S), anti-DNAJB8 (Abcam, ab235546), anti-GAPDH (Abcam, ab8245), and anti-P-gP (1:1000; Abcam, ab170904). Proteins were detected with the appropriate secondary antibodies conjugated to alkaline phosphatase by chemiluminescence (Amersham Pharmacia Corp, Piscataway, NJ, USA).

### Promotor-reporter gene construction

As previously described [15], to generate a luciferase reporter under the control of the MDR1 promoter, a 241-bp DNA fragment containing the MDR1 promoter from nucleotide (nt) −198 to +43 was amplified using forward primer 5′-GCGCTAGCCTAGAGAGGTGCAACG-3′ and reverse primer 5′-GCAGATCTGCGGCCTCTGCTTCTT-3′. The PCR product was cloned into a pGL3-Basic vector and confirmed by DNA sequencing.

### Immunoprecipitation

Cell lysates were centrifuged for 20 min at 12,000 rpm and 500 μl of the supernatant was transferred to another tube. 3 μg of IgG (Santa Cruz, CA, USA) and primary antibodies, together with 50 μl A/G-Agarose (Santa Cruz, CA, USA), were added into the cell lysate and incubated at 4 °C for 12 h. After this incubation, the samples were centrifuged and the beads were washed with RIPA. The remaining beads were added to 20 μl of RIPA buffer with 5× sample buffer, denatured at 95 °C for 10 min, and used for western blot assays.

### Luciferase reporter gene assays

An MDR1 luciferase report gene vector with an MDR1 consensus oligonucleotide was constructed as previously described [15]. Briefly, to generate a luciferase reporter under the control of the MDR1 promoter, a 241-bp DNA fragment containing the MDR1 promoter from nucleotide (nt) −198 to +43 was amplified using forward primer 5′-GCGCTAGCCTAGAGAGGTGCAACG-3′ and reverse primer 5′-GCAGATCTGCGGCCTCTGCTTCTT-3′. The PCR product was cloned into a pGL3-Basic vector and confirmed by DNA sequencing. And then, the luciferase report gene vector was transfected into colon cancer cells. After 48 h incubation, the luciferase activity was detected and calculated to evaluate MDR1 binding activity.

### Immunofluorescence microscopy

Immunofluorescence analysis was performed as described previously [16]. Cells was washed twice with PBS, the cells were fixed with 4% paraformaldehyde for 20 min at room temperature, permeabilized for 10 min with 0.2% Tri-ton X-100 (Sigma), and blocked with 5% BSA in PBST (phos-phate-buffered saline plus Tween 20) for 1 h. Before subjecting to secondary antibodies (Jackson ImmunoResearch) for 1 h at room temperature, the cells were incubated with the primary antibodies overnight at 4 °C and washed with PBST three times. Confocal laser scanning of fixed cells was detected using a Zeiss laser scanning microscope.

### AlphaLISA assay

We designed an AlphaLISA assay (PerkinElmer) to specifically measure DNAJB8–TP53 interactions in a microplate format. We used a monoclonal mouse anti-DNAJB8 antibody (Abcam, ab235546) conjugated to AlphaLISA acceptor beads (PerkinElmer). We used a monoclonal mouse biotinylated anti-P53 antibody (Cell Signaling Technology, 2524 S) to capture streptavidin-coated donor beads using AlphaScreen (PerkinElmer). Plasma samples of 5 μl were diluted with 5 μl of assay buffer (25 mM HEPES, pH 7.4, with 0.1% BSA, 0.05% Tween, and 100 mM NaCl) and incubated with 10 μl of anti-P53 acceptor beads (10 μg/ml) plus 10 μl of biotinylated anti-DNAJB8 antibody (1 nM) for 1 h at 25 °C. Subsequently, 20 μl of the streptavidin-coated donor beads (40 μg/ml) was added, and samples were incubated for 30 min. Plates were read on an EnSpire Multimode Microplate reader.

### Immunohistochemical staining and assessment

Immunohistochemical analysis (IHC) was performed as previously described [17]. In brief, colon cancer sections were subjected to deparaffinization, antigen retrieval and blockage of non-specific binding. The sections were then incubated with P53 (Cell Signaling Technology, 2524 S), DNAJB8 (Abcam, ab235546) and P-gP antibodies (Abcam, ab170904) at 4 °C overnight followed by incubation with a biotinylated secondary antibody and 3,3′-diaminobenzidine (Sigma). Immunostaining assessment was determined using composite scores by multiplying the percentage of immunoreactive cells (10–25% as 1, 26–50% as 2, 51–75% as 3 and 76–100% as 4) and the staining intensity (no staining as 0, weak staining as 1, moderate staining as 2, and strong staining as 3). The final staining score was divided into a low expression group (≤ 6) and a high expression group (> 7).

### In vivo drug efficacy

Tumors were established by subcutaneous injection of cancer cells into the dorsal flank of 5-week-old male BALB/c Nu/Nu mice. For SW620 tumor xenografts, 1 × 10^6^ cells were injected. For SW480 tumor xenografts, 2.5 × 10^6^ cells were injected. Treatment began when tumors reached 100 to 150 mm^3^. Mice were weighed and randomly sorted into treatment groups (8 mice per group). L-OHP (20 mg/kg) was administered via tail-vein intravenous (IV) injection into tumors every three days for 21 d. For in vivo sEVs treatment, sEVs were injected intratumorally twice a week (5 μg sEVs per injection).

The vehicle alone was used for the negative control group. Measurements were recorded every 3 days using a digital caliper. Tumor volumes were estimated by measuring two perpendicular diameters, a and b, using the formula V = 0.5 × a × b^2^, where a and b indicate the long and short perpendicular diameters, respectively.

Non-invasive bioluminescence imaging was performed by using the IVIS Lumina System (PerkinElmer, Waltham, MA, USA). Briefly, mice under isoflurane anaesthesia were injected with 100 mg/kg D-Luciferin in 200 μl PBS into the peritoneal cavity and, 5 min after injection, imaged with the IVIS Lumina System. The total peak bioluminescent signal intensities were calculated using Living Image 4.0 software (PerkinElmer, Waltham, MA, USA).

Animal care and experimental procedures were approved by the Ethics Committee in Animal Experimentation of West China Hospital, Sichuan University, Chengdu, China (record #: 2018192 A).

### Statistical analysis

All of the statistical analyses were performed using the SPSS statistical program (Chicago, IL, USA). Data were presented as mean ± SD of at least three independent tests. Correlations between parameters were assessed using the Pearson correlation analysis. Student’s t-test was used to compare the two groups. Values of *P* < 0.05 were considered significant. The association between protein expression and clinicopathological parameters was analyzed with the χ^2^ test. For survival analyses, Kaplan–Meier survival curves were constructed and differences were tested by the log-rank test. Univariate and multivariate Cox proportional hazards regression was carried out to determine the effects of the clinicopathological variables and DNAJB8 expression on the patient survival. Nomograms were generated according to the Cox regression coefficients of selected variables, and the predictive accuracy of every nomogram was evaluated with calibration plots. Nomogram and calibration plots were generated using R in R Studio (Version 1.1.447). *p* values less than 0.05 were considered statistically significant.

## Results

### DNAJB8 is overexpressed in L-OHP-resistant COAD cells

**Fig. 1:**
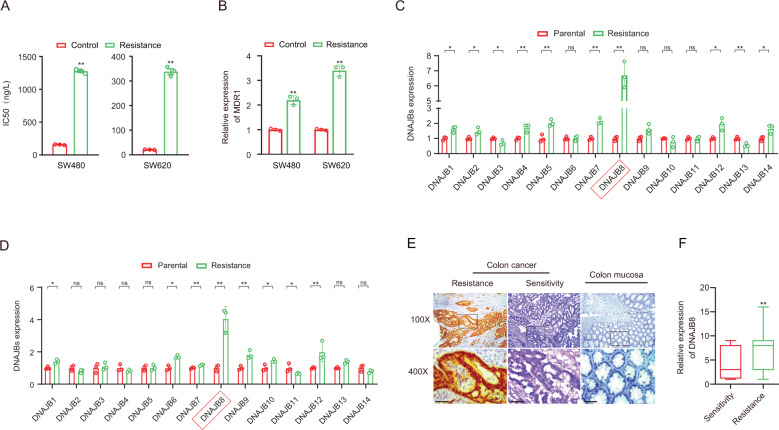
Overexpression of DNAJB8 in L-OHP-resistant COAD cells. **A** IC_50_ Values of L-OHP in COAD cells treated with L-OHP. **B** Expression levels of MDR1(P-glycoprotein) were determined by qRT-PCR in L-OHP-resistant COAD cells. Expression levels of DNAJBs were determined by qRT-PCR in L-OHP-resistant SW480 **C** and SW620 **D** cells. Experiments were repeated three times and representative results are shown. **E** Expression analysis of DNAJB8 protein in tissues from COAD patients treated with L-OHP therapy by immunohistochemistry. **F** DNAJB8 expression was analyzed in responding and nonresponding groups of patients. Results shown are mean ± s.d. from a representative experiment. *p < 0.05; **p < 0.01; Student’s *t* test. Similar results were obtained in three independent experiments.

The chemoresistant cell model (SW620/L-OHP and SW480/L-OHP) was established from the human colorectal adenocarcinoma cell line SW620 and SW480 through L-OHP induction (Fig. 1A). Our data showed that mRNA and protein expression of the superfamily of the ATP-binding cassette transporter family member P-glycoprotein (P-gp) was upregulated in chemoresistant cells compared with parental cells (Fig. 1B), which indicated that the establishment of the L-OHP-resistant colorectal cancer cell line was successful.

Our previous data showed that the DNAJB protein family is overexpressed in COAD cells [18]. To elucidate the mechanisms underlying the L-OHP resistance in COAD cells, we evaluated the expression of all DNAJB family proteins in SW620/L-OHP and SW480/L-OHP cells. DNAJB8 was the most strongly expressed DNAJB in L-OHP-resistant cells compared with parental cells (Figs. 1C, S1).

To study the prevalence and clinical significance of DNAJB8 overexpression in COAD L-OHP resistance, we quantified the expression of DNAJB8 by immunohistochemistry (IHC) in a cohort of 220 COAD patients who were treated with postoperative L-OHP chemotherapy. Patients were divided into responding and nonresponding groups according to the response evaluation criteria. We found that the DNAJB8 expression level was much higher in nonresponding than in responding patients (Fig. 1D and E).

### DNAJB8 silencing sensitizes COAD cells to L-OHP chemotherapy

**Fig. 2:**
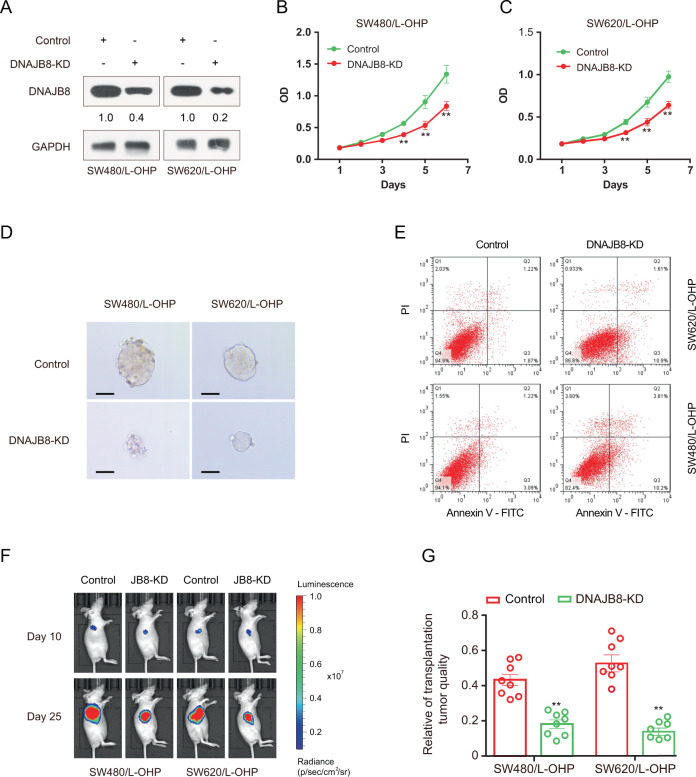
DNAJB8 silencing sensitizes COAD cells to L-OHP chemotherapy. **A** DNAJB8 expression was detected by western blot assay in L-OHP-resistant COAD cells following siRNA transfection. SW620/L-OHP and SW480/L-OHP cells with DNAJB8 knockdown were seeded for cell proliferation **B**, **C** mammosphere formation **D** and flow cytometry assay **E** Scale bars = 40 μm. Luminescence imaged tumor-bearing nude mice were injected with DNAJB8 knockdown L-OHP resistant cells and control cells **F**, and transplanted tumor volume was detected **G**. Results shown are mean ± s.d. from a representative experiment. *p < 0.05; **p < 0.01; Student’s *t* test. Similar results were obtained in three independent experiments.

To examine the biological relevance of DNAJB8 as a molecule that is potentially involved in chemoresistance in COAD, we assessed the effects of DNAJB8 silencing on the sensitivity of cells exposed to L-OHP. We established DNAJB8 knockdown L-OHP-resistant COAD cells using specific small interfering RNA (siRNA) (Fig. 2A). Interestingly, our results showed that downregulation of DNAJB8 re-sensitized SW620/L-OHP and SW480/L-OHP cells to L-OHP treatment, as shown by the growth curve, mammosphere formation assays, and increased apoptosis rates (Fig. 2B–E). These results indicate that DNAJB8 can modulate the L-OHP-resistant phenotype of COAD cells.

Our data showed that MDR1 expression was upregulated in the chemoresistant cells. Next, we examined the effects of DNAJB8 knockdown on MDR1 expression. Our results showed that DNAJB8 knockdown significantly decreased MDR1 expression at both the transcript and protein levels in SW620/L-OHP cells. Similar results were observed in SW480/L-OHP cells (Fig. S2A, B). These data indicate that the contribution of P-gP is very important for DNAJB8-mediated L-OHP resistance in COAD.

To verify the effect of DNAJB8 on L-OHP-exposed colon tumor in vivo, we assessed tumorigenicity of DNAJB8 knockdown L-OHP-resistant cells in a nude mouse model. Tumor xenografts of DNAJB8 knockdown SW620/L-OHP and SW480/L-OHP cells were markedly more sensitive to L-OHP compared with their counterparts after 2 weeks, and a similar trend was maintained until study termination (Figs. 2F-G and S2C-D). These observations also suggest that DNAJB8 is involved in modulating the sensitivity of COAD cells to L-OHP.

### DNAJB8 promotes TP53 stability in L-OHP-resistant COAD cells

**Fig. 3:**
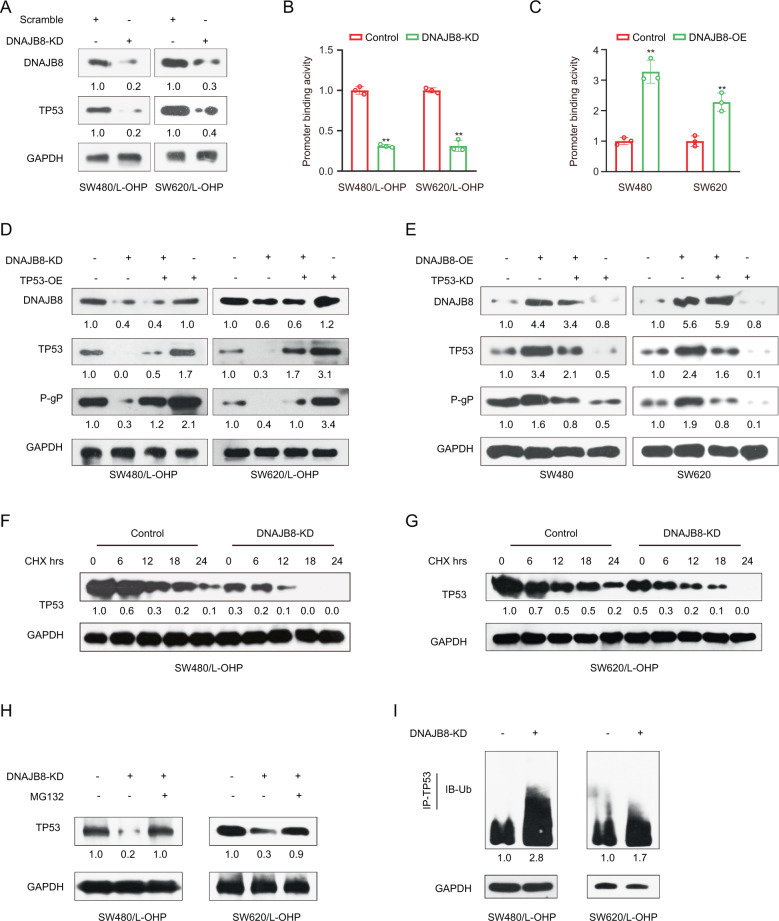
DNAJB8 interacts with TP53 in L-OHP-resistant COAD cells. **A** Expression of TP53 was analyzed by western blot assay in L-OHP-resistant COAD cells transfected with DNAJB8 specific siRNA. MDR1 promoter binding activity by TP53 was detected in L-OHP-resistant colon cancer cells **B** and parental colon cancer cells **C** using a luciferase reporter gene assay. **D** Restoration of DNAJB8 knockdown L-OHP-resistant colon cancer cells using a TP53 (R273H) overexpression vector. Indicated proteins were detected by western blot assay. **E** Restoration of DNAJB8 overexpression L-OHP-resistant colon cancer cells using a TP53 (R273H) specific siRNA. Indicated proteins were detected by western blot assay. L-OHP-resistant SW480 **F** and SW620 **G** cells transfected with DNAJB8-specific siRNA were treated with cycloheximide (CHX) (20 μg/ml) for the indicated time. **H** L-OHP-resistant colon cancer cells transfected with DNAJB8-specific siRNA were treated with MG132. **I** TP53 ubiquitylation expression was detected in DNAJB8 knockdown L-OHP-resistant colon cancer cells by western blot assay. Results shown are mean ± s.d. from a representative experiment. *p < 0.05; **p < 0.01; Student’s *t* test. Similar results were obtained in three independent experiments.

Previous data showed that HSP40 family proteins could affect the protein expression of P53 in breast cancer and COAD cells [19, 20]. Based on an increasing body of evidence suggesting that MDR1 is involved in the TP53 signaling pathway as one of the target genes in several cancers, including COAD [21], we measured the expression and activity of the TP53 in SW480 and SW620 cell lines expressing the mutant R273H P53. Interestingly, the mRNA expression of TP53 was not affected (Fig. S3A), but the protein expression of TP53 were was substantially downregulated when DNAJB8 was knocked down in SW480/L-OHP and SW620/L-OHP cells (Fig. 3A). Similar results were obtained using MDR1, ID2 [22], and CXCL5 [23] promoter vectors with respect to the DNA-binding ability of TP53 (Figs. 3B and S3B-C). Moreover, ectopic expression of DNAJB8 remarkably increased the DNA-binding ability of TP53 in parental cells (Fig. 3C), further supporting that TP53 abundance is positively controlled by DNAJB8. Results from rescue experiments showed that TP53 (R273H) upregulation significantly rescued MDR1 expression and restored L-OHP sensitivity after DNAJB8 knockdown in L-OHP-resistant cells (Fig. 3D). Furthermore, TP53 (R273H) inhibition significantly reduced the downregulation of MDR1 expression and L-OHP resistance after DNAJB8 upregulation in parental cells (Fig. 3E). These data suggested that DNAJB8 promotes L-OHP resistance in a TP53-dependent manner.

To determine whether DNAJB8 regulates TP53 protein stability, we treated L-OHP-resistant cells with cycloheximide (CHX) to block de novo protein synthesis and then evaluated the rate of TP53 degradation. Our data showed that the rate of TP53 degradation in DNAJB8 knockdown cells was significantly higher than that in control cells (Fig. 3F, G), suggesting that DNAJB8 may control TP53 protein levels. Together, these results suggest that DNAJB8 positively regulates TP53 stability at the post-translational level. Considering TP53 is a labile protein degraded by the proteasome, we speculated that DNAJB8 may affect TP53 stability via the ubiquitination degradation pathway. Indeed, TP53 protein expression did not decrease further when DNAJB8 knockdown SW480/L-OHP and SW620/L-OHP cells were treated with the proteasome inhibitor MG132 (Fig. 3H), suggesting that DNAJB8 silencing promotes TP53 degradation by the ubiquitination pathway. We also performed immunoprecipitation assays and showed that TP53 polyubiquitination was increased when DNAJB8 was inhibited (Fig. 3I), indicating that DNAJB8 inhibits TP53 degradation through the ubiquitination pathway.

### DNAJB8 interacts with TP53 in L-OHP-resistant COAD cells

**Fig. 4:**
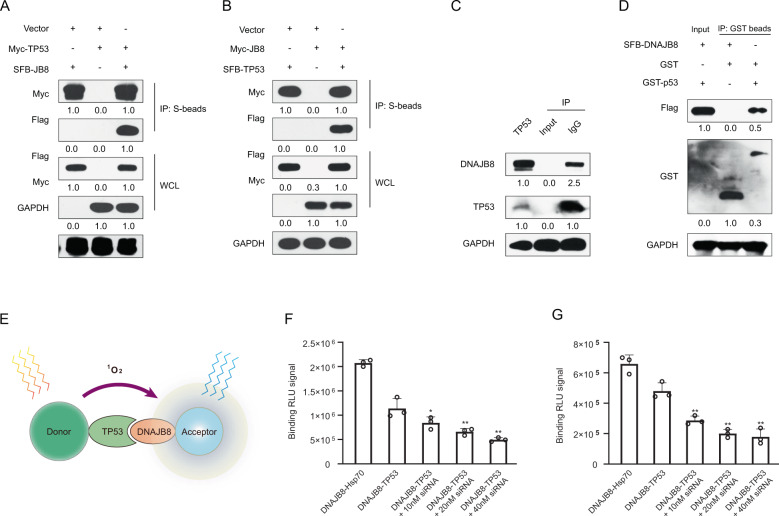
DNAJB8 directly interacts with TP53 in vivo and in vitro. **A** HEK293T cells transfected with the indicated constructs were collected 24 h later. Cells were lysed with NETN buffer. **C** Immunoprecipitation (IP) using S-protein agarose was performed and the western blotted was done with the indicated antibodies. **B** Endogenous DNAJB8 associated with TP53 was analyzed using SW480/L-OHP cells. **D** Beads coated with GST or GST-TP53 fusion proteins were incubated with SFB-DNAJB8 protein overnight. GST pulldown was immunoblotted with the indicated antibodies. **E** The design of the AlphaLISA assay for DNAJB8–TP53 interactions. The binding signal expressed in binding relative luminescence units (RLUs) between DNAJB8 and TP53 in SW480/L-OHP cells **F** and SW620/L-OHP cells **G**. Results shown are mean ± s.d. from a representative experiment. *p < 0.05; **p < 0.01; Student’s *t* test. Similar results were obtained in three independent experiments.

Recent studies have indicated that the HSP40 protein DNAJA1 directly interacts with mutant P53 and stabilizes mutant P53 [19]. To elucidate how DNAJB8 controls TP53 stability, we first investigated whether DNAJB8 interacts with TP53 in cells. As expected, we confirmed the co-localization of DNAJB8 and TP53 in L-OHP resistant cancer cells using confocal microscopy (Fig. S4). Moreover, exogenously expressed DNAJB8 co-immunoprecipitated with TP53 and vice versa (Fig. 4A). To further confirm this interaction, we performed an endogenous co-immunoprecipitation experiment and detected a complex containing DNAJB8 and TP53 (Fig. 4B). Importantly, we found that recombinant GST-tag-expressed TP53 (R273H) immunoprecipitated with ectopically expressed DNAJB8 (Fig. 4C), indicating that DNAJB8 directly associates with TP53 in vitro. In order to confirm the direct interaction between DNAJB8 and TP53, an AlphaLISA assay was carried out for the specific detection of DNAJB8–TP53 interactions in SW480/L-OHP and SW620/L-OHP cells (Fig. 4D). Our data showed that the endogenous DNAJB8–TP53 interactions in DNAJB8 knockdown SW480/L-OHP and SW620/L-OHP cells were significantly decreased (*P* < 0.01) (Fig. 4E, F). Together, these data strongly suggest that DNAJB8 directly binds to TP53 in vivo and in vitro.

### DNAJB8 positively correlates with TP53/MDR1 pathway expression in COAD

**Fig. 5:**
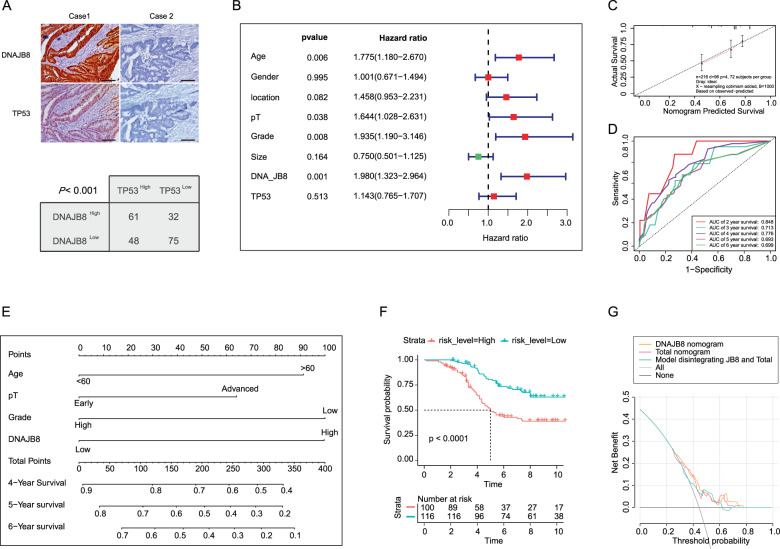
DNAJB8 positively correlates with TP53/P-gP pathway expression in COAD. **A** DNAJB8 and TP53 expressions were tested using IHC in the COAD tissues. **B** The results of multivariate COX regression analysis were shown in a tree diagram. **C** The calibration curve for predicting patient survival at 5 years; the nomogram-predicted probability of overall survival is plotted on the x-axis; and the actual overall survival is plotted on the y-axis. **D** The results of the receiver operating characteristic (ROC). **E** Prognostic nomogram for COAD. **F** Kaplan–Meier curve analyses. **G** Decision curve analysis for the prognostic nomogram, where the y-axis measures the net benefit. Results shown are mean ± s.d. from a representative experiment. *p < 0.05; **p < 0.01; Student’s *t* test. Similar results were obtained in three independent experiments.

Based on the experimental results above, we hypothesized that DNAJB8 and the TP53/MDR1 pathway might be co-expressed in COAD tissues. IHC results showed that DNAJB8 and TP53 co-localized to the nuclei of colon tumor cells. Statistical analysis showed a significant positive correlation between DNAJB8 and TP53 expression in the COAD tissues tested (Fig. 5A and Table S2, *P* < 0.001). Next, we determined the significant predictive variables through univariate Cox regression analysis. Variables with *P*-value < 0.05 in the univariate analysis were included in the multivariate model (Fig. 5B), which included the following predictive factors: age, pT, grade, and DNAJB8 levels. A nomogram model was built using the coefficients of the multivariate Cox regression model (Fig. 5E and Table S3). Surprisingly, the C-index of the nomogram for predicting survival was 0.841 (95% confidence interval, 0.802–0.880). According to the calibration curve, predictive values were consistent with observed values considering the probabilities of 3-, 5-, and 7-year overall survival (OS) (Fig. 5C). The area under the ROC curve (AUC) values for 2-, 3-, and 4-year survival using the predictive nomogram were 0.848, 0.713, and 0.776, respectively (Fig. 5D). Finally, we calculated the total risk score based on each predictor in the nomogram model. Kaplan–Meier analysis showed that patients with a high risk score had an obviously worse OS than patients with a low risk score (*P* < 0.001, log-rank test; Fig. 5F).

### DNAJB8 levels in serum correlate with L-OHP response in COAD patients

To examine whether DNAJB8 could be present in the extracellular milieu, we analyzed DNAJB8 levels in the serum of COAD patients and the CM of COAD cells using ELISA. As shown in Fig. S5A, the serum DNAJB8 levels were higher in COAD patients compared with healthy donors. Moreover, serum DNAJB8 levels decreased after tumor resection (Fig. S5B), indicating that serum DNAJB8 was mainly produced by COAD cells. Consistent with the upregulation of DNAJB8 in L-OHP-resistant cells, DNAJB8 levels were significantly higher in the CM of resistant cells than in that of parental cells (Fig. S5C).

We next explored whether circulating DNAJB8 could predict the response to L-OHP in COAD patients. As shown in Fig. S5D, the average level of DNAJB8 in pre-therapy serum was higher in nonresponders than in responders. Moreover, a prognostic signature based on DNAJB8 expression in serum has been built. Patients with high risk scores exhibited significantly worse OS than patients with low risk scores (Fig. S5E–I).

### DNAJB8 transfer by small extracellular vesicles was confirmed in COAD

**Fig. 6:**
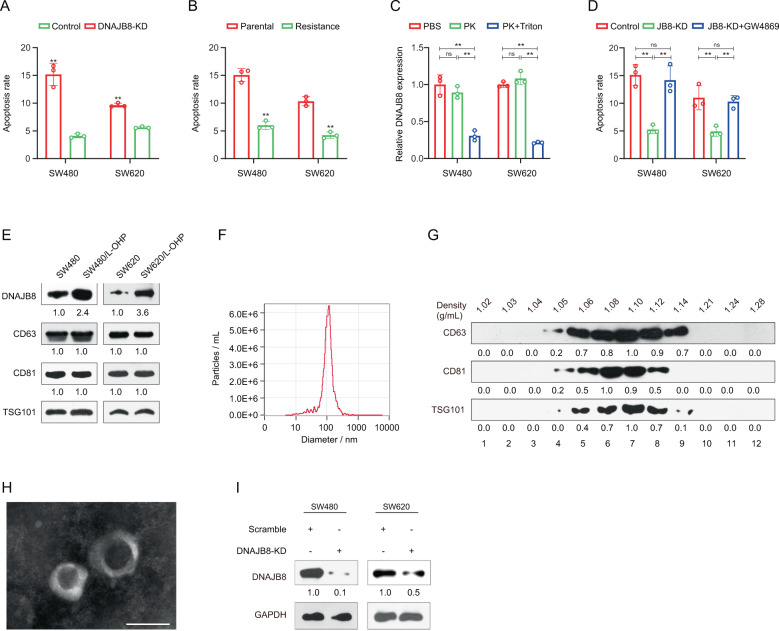
DNAJB8 transfer by small extracellular vesicles confirmed in colon cancer. **A** The apoptosis rate was detected in parental cells cocultured with culture medium (CM) from L-OHP resistant cells and DNAJB8-knockdown-resistant cells. **B** The apoptosis rate was detected in parental cells cocultured with parental cells and L-OHP resistant cells. **C** DNAJB8 expression was analyzed using ELISA assay in the CM of L-OHP cells treated with Proteinase K (PK) alone or combined with Triton X-100 for 20 min. **D** DNAJB8 expression was analyzed using ELISA assay in the CM of DNAJB8 knockdown L-OHP cells treated with GW4869. **E** DNAJB8 and sEVs biomarker expression in sEVs from colon cancer cells were detected using by western blot assay. **F** The sizes of sEVs from colon cancer cells were verified using the NTA method. **G** CD63, CD81, and TSG101 expression was detected in fractions collected from OptiPrepTM density gradient centrifugation by western blot assay. (H) sEVs obtained from fraction 7 (density 1.10 g/mL) were verified using transmission electron microscopy. Scale bar = 200 nm. Results shown are mean ± s.d. from a representative experiment. *p < 0.05; **p < 0.01; Student’s *t* test. Similar results were obtained in three independent experiments.

Previous data showed that chemoresistant cells could regulate chemotherapy sensitivity of parental cells through autocrine or paracrine secretion. In our system, interestingly, we found that L-OHP resistance in parental cells increased upon culturing with CM from SW480/L-OHP and SW620/L-OHP cells, but not upon culturing with CM from DNAJB8 knockdown L-OHP-resistant cells (Fig. 6A). Similar results were obtained when culturing parental cells with CM from SW480/L-OHP and SW620/L-OHP cells (Fig. 6B). These results indicated that extracellular DNAJB8 was transferred to parental cells, promoting L-OHP resistance of COAD cells.

Next, the expression pattern of extracellular DNAJB8 was investigated in L-OHP-resistant cells. The levels of DNAJB8 in CM were unchanged upon Proteinase K treatment but significantly decreased when treated using Proteinase K and Triton X-100 simultaneously (Fig. 6C). Moreover, the EV inhibitor neutral sphingomyelinase 2 (nSMase2, GW4869) was used to treat L-OHP-resistant cells before culturing parental cells with CM. There was no difference in L-OHP resistance between L-OHP-resistant cells and parental cells (Fig. 6D). All these data indicated that extracellular DNAJB8 was mainly wrapped by EVs instead of being directly released.

We next extracted sEVs from COAD cells. Using a Western blot assay, we showed that the sEV protein markers CD63, CD81, and TSG101 were highly expressed in sEVs from COAD cells (Fig. 6E). Next, we measured the sizes of sEVs by the NTA method and found that the sEVs were sized between 40 and 150 nm (Fig. 6F), in agreement with previously reported sEVs sizes. Calnexin, which is not present in sEVs, was not observed after Western blot assay (Fig. S6). In addition, sEVs were purified using an OptiPrep™ density gradient. Fractions of increasing density were collected, and Western blot analysis was performed to identify sEVs in enriched samples. As shown in Fig. 6G, sEVs were enriched in fractions 6–8, corresponding to a density of 1.08–1.12 g/mL. This density is consistent with previously reported studies conducted on different cell types. Next, transmission electron microscopy analysis of sEVs obtained from fraction 7, corresponding to a density of 1.10 g/mL, revealed vesicles that were consistent with sEVs in size and morphology (Fig. 6H). Thus, we successfully extracted sEVs secreted by COAD cells. As expected, DNAJB8 levels were significantly higher in sEVs from L-OHP-resistant COAD cells than in those from parental cells (Fig. 6I).

### Intercellular transfer of DNAJB8 by sEVs increases L-OHP resistance

**Fig. 7:**
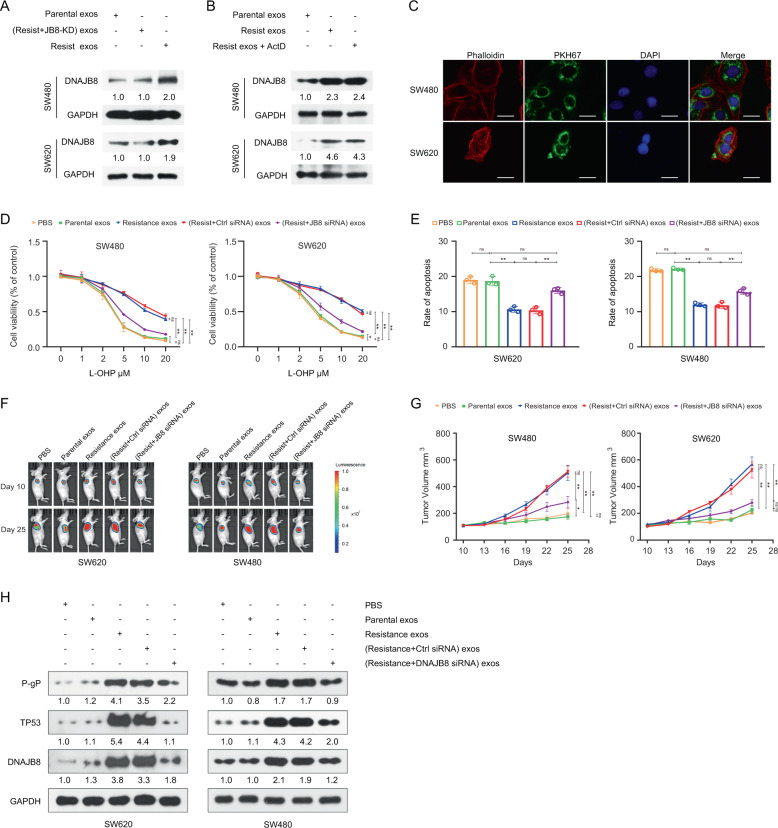
Intercellular Transfer of DNAJB8 by sEVs increases L-OHP Resistance. **A** DNAJB8 expression was detected by western blot assay in parental cells incubated with sEVs from resistant cells. **B** DNAJB8 expression was detected by western blot assay in parental cells incubation with sEVs from resistant cells treated with actinomycin D (ActD). **C** Parental cells were incubated directly with sEVs from L-OHP resistant cells. Scale bar = 40 µm. **D** The apoptosis and proliferation **E** were evaluated in parental cells incubated with sEVs from L-OHP resistant cells. **F** Subcutaneous xenograft assay of SW480 and SW620 cells in nude mice with intratumoral injection of indicated exosomes upon L-OHP treatment. **G** Volumes of tumors are shown (*n* = 5 per group). **H** TP53 and P-gP expression were detected in parental COAD cells incubated with sEVs from L-OHP resistant cells. Results shown are mean ± s.d. from a representative experiment. *p < 0.05; **p < 0.01; Student’s *t* test. Similar results were obtained in three independent experiments.

sEVs derived from cancer cells associated with the transfer of noncoding RNA and cancer-promoting proteins can be internalized by neighboring cells [24, 25]. As expected, the intracellular levels of DNAJB8 were increased upon incubation with sEVs from L-OHP-resistant cells, but not upon incubation with sEVs from DNAJB8 knockdown L-OHP-resistant cells (Fig. 7A). The increase of DNAJB8 levels in recipient cells was not affected by the RNA polymerase II inhibitor actinomycin D, excluding the involvement of endogenous induction (Fig. 7B). To further confirm that DNAJB8 could be transferred to recipient cells via sEVs, we isolated and labeled sEVs with PKH67. As shown in Fig. 7C, after incubation with labeled sEVs, co-localization of PKH67 and COAD cells was observed in recipient cells. We further examined whether sEV-transferred DNAJB8 could confer the resistant phenotype to recipient COAD cells. In a co-culture mammosphere formation, apoptosis, and proliferation assay, parental cells incubated directly with sEVs from L-OHP-resistant cells displayed reduced sensitivity to L-OHP (Fig. 7D), which could be abrogated by treatment with siRNA against DNAJB8 in recipient cells. Parental COAD cells incubated with sEVs from resistant cells displayed increased expression of P53 and P-gP, which was abrogated by treatment with siRNA against DNAJB8 in recipient cells (Fig. 7E). It has been reported that P-gP could be packaged into the sEVs of breast cancer cells. However, in the present study P-gP expression in resistant cells was not significantly different from that in parental cells (Fig. S7). Taken together, these data indicated that the transfer of exosomal DNAJB8 confer·red L-OHP resistance to the recipient cells.

To demonstrate the effect of DNAJB8 from sEVs on the response to L-OHP in vivo, we administered sEVs derived from L-OHP-resistant and parental cells intratumorally into COAD cell xenografts. Our data showed that sEVs from L-OHP-resistant cells significantly dampened the response of COAD xenografts to L-OHP, but those from parental cells did not (Fig. 7F and G), accompanied by increased DNAJB8 expression in the tumors (Fig. 7H). Our data suggested that DNAJB8 might modulate L-OHP resistance through the P53/MDR1 signaling pathway in vivo.

**Fig. 8:**
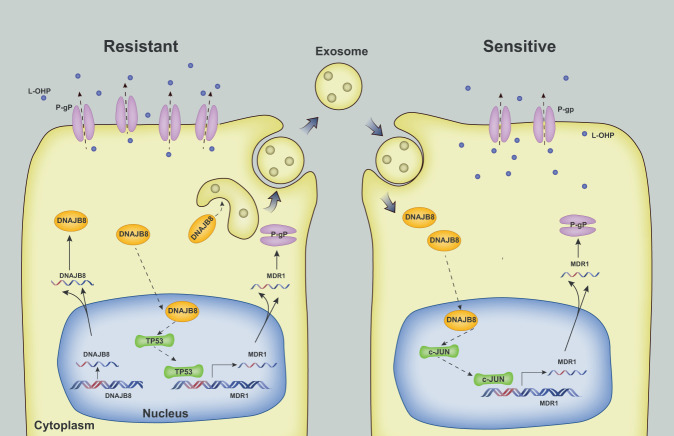
A schematic diagram of sEVs DNAJB8 based signaling pathway in CRC L-OHP resistance. In parental cells, sEV-mediated transfer of DNAJB8 derived from L-OHP resistant COAD cells promoted L-OHP resistance by binding and inhibiting ubiquitination of TP53, leading to the increased expression of P-gP and upregulation of the L-OHP efflux.

Next, a prognostic signature based on the expression of sEV-transferred DNAJB8 in serum has been built. COAD patients with high risk scores exhibited significantly worse OS than patients with low risk scores (Fig. S8). Collectively, these findings indicated that sEVs from resistant cells could endow recipient COAD cells with L-OHP resistance via intercellular transfer of DNAJB8, conferring L-OHP resistance (Fig. 8).

## Discussion

L-OHP-based chemotherapy remains one of the most widely used COAD treatments. In the past, much attention has been paid to the mechanisms underlying L-OHP resistance [3, 26]. In the present study, we investigated the potential role of DNAJB8 in the response of COAD to L-OHP treatment and the underlying mechanisms.

As a first-line drug for COAD treatment, resistance develops after long-term L-OHP usage, which leads to refractory tumors [27, 28]. To investigate the mechanisms underlying L-OHP resistance in COAD, L-OHP-resistant COAD cell lines were established by continuous exposure of SW480 and SW620 cells to L-OHP from a low concentration to gradually increasing concentrations. We found that DNAJB8 is upregulated in SW480/L-OHP and SW620/L-OHP cells. DNAJB8 knockdown improves the response of COAD cells to L-OHP treatment both in vitro and in vivo, in a TP53-dependent manner. Furthermore, DNAJB8 could be incorporated into sEVs and transmitted to parental cells, thus promoting L-OHP resistance. Treatment of L-OHP-resistant COAD cells with siRNA targeting DNAJB8 restored the response to L-OHP.

In the present study, we demonstrated that DNAJB8 and P-gP are both upregulated in L-OHP-resistant COAD cells and tissues. Abnormal expression of CDC42, P-gP, and MRP1 can be induced in L-OHP-resistant human COAD cells [29, 30]. The SIRT1–NRF2 pathway is activated in L-OHP-resistant colon cell lines [30–32]. Moreover, L-OHP-resistant gastric cancer cells exhibit elevated expression of HSP72, and HSP72 overexpression is critical for L-OHP resistance [32]. Consistent with previous studies, our data showed that DNAJB8 silencing can promote sensitivity through upregulation of MDR1 expression in L-OHP-resistant COAD cells. The expression of MDR1, a molecule involved in multidrug resistance in COAD, may be related to the resistance to multiple chemotherapeutics including L-OHP and Fluorouracil. Our results showed that the resistance of colon cancer cells to both 5-FU increased following DNAJB8 upregulation (Fig. S9), which suggests that DNAJB8 may promote the multidrug resistance of COAD through MDR1. Activated ERK phosphorylates the transcription factor c-Jun, contributing to nuclear translocation of c-Jun and subsequently promoting MDR1 expression [33]. Additionally, luciferase reporter assays revealed the β-catenin-responsive elements in the promoter of the human *MDR1* gene [34, 35]. Nuclear translocation of c-Jun and β-catenin was not significantly different between L-OHP-resistant cells and parental cells (Fig. S10), which indicated that transcriptional activation of *MDR1* mainly relied on TP53 function.

A growing number of studies have provided compelling evidence that gain-of-function P53 mutants promote cancer development and progression. It has been reported that Ets-1 interacts with mutant P53, but not with wild-type P53, in vivo. Our results also show that DNAJB8 can interact with mutant TP53, but can weakly interact with wild-type P53 (Fig. S11), which may be due to changes in the spatial conformation of the P53 protein after mutation and the interactive abilities of different conformations of P53.

The regulation of P53 depends on a sophisticated chaperone machine composed of HSP70, HSP90, and HSP40. HSP70 was reported to promote the stability of wild-type P53 and maintain its DNA-binding ability. By inhibiting ubiquitination, stable association with HSP90 was shown to increase the half-life of mutant P53. Using quantitative reverse transcription-PCR (qRT-PCR) and Western blot analysis, we found that the expression of HSP70 and HSP90 in the L-OHP-resistant COAD cell lines was not significantly different compared with that in parental cells (Fig. S12), suggesting that DNAJB8 may directly bind to TP53 without depending on HSP70 and HSP90 to promote the stability of TP53.

Ganetespib, an HSP90 inhibitor, modulates DNA methylation through downregulation of DNMT expression to inhibit COAD proliferation and survival [36]. Pharmacological inhibition of HSP70 with VER-155008 can induce caspase-3/7-dependent apoptosis in COAD cells [18]. While our work has provided invaluable insight into the role of DNAJB8 in L-OHP-resistant COAD, ongoing research is needed to confirm whether inhibitors of DNAJB8 could potentially promote L-OHP sensitivity in COAD and to determine possible underlying mechanisms.

sEV transport has been reported to play an important role in modulating cell signaling and biological function in recipient cells [37]. sEVs, which transfer noncoding RNAs and drug efflux pumps, might confer drug resistance traits from drug-resistant cells to drug-sensitive cells [38]. However, the contribution of exosomal proteins to the regulation of L-OHP resistance remains poorly understood. Here, our results suggest that DNAJB8 protein embedded in sEVs derived from L-OHP-resistant cells could confer the resistant phenotype to recipient COAD cells. Carcinoma-associated fibroblast-derived exosomal Wnt3α could induce the dedifferentiation of cancer cells to promote L-OHP resistance in COAD [39]. However, sEVs from normal intestinal cells could increase chemosensitivity of L-OHP-resistant COAD [40].

Personalized medicine is the current trend in chemotherapy, and the identification of novel potential therapeutic targets is a major challenge. Here, we systematically explored the effects of DNAJB8 knockdown on L-OHP sensitivity in cells and cell line-based xenografts, suggesting this treatment approach could be used for L-OHP-resistant COAD.

## Supplementary information

aj-checklist-DNAJB8

supplementary files

Raw data of WB

## Data Availability

Original data and code used in this study are provided in the supplementary file. All data is accessible.

## References

[1] Siegel RL, Miller KD, Jemal A (2018). Cancer statistics, 2018. CA Cancer J Clin.

[2] Brenner H, Kloor M, Pox CP (2014). Colorectal cancer. Lancet.

[3] Dienstmann R, Salazar R, Tabernero J (2015). Personalizing colon cancer adjuvant therapy: selecting optimal treatments for individual patients. J Clin Oncol.

[4] Théry C, Zitvogel L, Amigorena S (2002). Exosomes: composition, biogenesis and function. Nat Rev Immunol..

[5] Théry C, Ostrowski M, Segura E (2009). Membrane vesicles as conveyors of immune responses. Nat Rev Immunol..

[6] EL Andaloussi S, Mäger I, Breakefield XO, Wood MJ (2013). Extracellular vesicles: biology and emerging therapeutic opportunities. Nat Rev Drug Disco.

[7] Hageman J, Rujano MA, van Waarde MA, Kakkar V, Dirks RP, Govorukhina N (2010). A DNAJB chaperone subfamily with HDAC-dependent activities suppresses toxic protein aggregation. Mol Cell.

[8] Wu J, Liu T, Rios Z, Mei Q, Lin X, Cao S (2017). Heat shock proteins and cancer. Trends Pharmacol Sci..

[9] Nillegoda NB, Wentink AS, Bukau B (2018). Protein disaggregation in multicellular organisms. Trends Biochem Sci..

[10] Nishizawa S, Hirohashi Y, Torigoe T, Takahashi A, Tamura Y, Mori T (2012). HSP DNAJB8 controls tumor-initiating ability in renal cancer stem-like cells. Cancer Res..

[11] Kusumoto H, Hirohashi Y, Nishizawa S, Yamashita M, Yasuda K, Murai A (2018). Cellular stress induces cancer stem-like cells through expression of DNAJB8 by activation of heat shock factor 1. Cancer Sci..

[12] Liang JW, Shi ZZ, Zhang TT, Hao JJ, Wang Z, Wang XM (2013). Analysis of genomic aberrations associated with the clinicopathological parameters of rectal cancer by array‑based comparative genomic hybridization. Oncol Rep..

[13] Mathivanan S, Fahner CJ, Reid GE, Simpson RJ (2012). ExoCarta 2012: database of exosomal proteins, RNA and lipids. Nucleic Acids Res..

[14] Chen JF, Luo X, Xiang LS, Li HT, Zha L, Li N (2016). EZH2 promotes colorectal cancer stem-like cell expansion by activating p21cip1-Wnt/β-catenin signaling. Oncotarget.

[15] Ogretmen B, Safa AR (1999). Negative regulation of MDR1 promoter activity in MCF-7, but not in multidrug resistant MCF-7/Adr, cells by cross-coupled NF-kappa B/p65 and c-Fos transcription factors and their interaction with the CAAT region. Biochemistry.

[16] Zhang Y, Feng YB, Shen XM, Chen BS, Du XL, Luo ML (2008). Exogenous expression of Esophagin/SPRR3 attenuates the tumorigenicity of esophageal squamous cell carcinoma cells via promoting apoptosis. Int J Cancer.

[17] Zhang Y, Du XL, Wang CJ, Lin DC, Ruan X, Feng YB (2012). Reciprocal activation between PLK1 and Stat3 contributes to survival and proliferation of esophageal cancer cells. Gastroenterology.

[18] Zhang TT, Jiang YY, Shang L, Shi ZZ, Liang JW, Wang Z (2015). Overexpression of DNAJB6 promotes colorectal cancer cell invasion through an IQGAP1/ERK-dependent signaling pathway. Mol Carcinog..

[19] Tong, X, Xu, D, Mishra, RK, Jones, RD, Sun, L, Schiltz, GE et al. Identification of a druggable protein-protein interaction site between mutant p53 and its stabilizing chaperone DNAJA1. J. Biol. Chem. (2020). 10.1074/jbc.RA120.014749 PMC7948449 33208462

[20] Tracz-Gaszewska Z, Klimczak M, Biecek P, Herok M, Kosinski M, Olszewski MB (2017). Molecular chaperones in the acquisition of cancer cell chemoresistance with mutated TP53 and MDM2 up-regulation. Oncotarget.

[21] Yan W, Chen X (2009). Identification of GRO1 as a critical determinant for mutant p53 gain of function. J Biol Chem..

[22] Yan W, Liu G, Scoumanne A, Chen X (2008). Suppression of inhibitor of differentiation 2, a target of mutant p53, is required for gain-of-function mutations. Cancer Res..

[23] Yeudall WA, Vaughan CA, Miyazaki H, Ramamoorthy M, Choi MY, Chapman CG (2012). Gain-of-function mutant p53 upregulates CXC chemokines and enhances cell migration. Carcinogenesis.

[24] Melo SA, Sugimoto H, O’Connell JT, Kato N, Villanueva A, Vidal A (2014). Cancer exosomes perform cell-independent microRNA biogenesis and promote tumorigenesis. Cancer Cell.

[25] Qu L, Ding J, Chen C, Wu ZJ, Liu B, Gao Y (2016). Exosome-transmitted lncARSR promotes sunitinib resistance in renal cancer by acting as a competing endogenous RNA. Cancer Cell.

[26] Grothey A, Sobrero AF, Shields AF, Yoshino T, Paul J, Taieb J (2018). Duration of adjuvant chemotherapy for stage III colon cancer. N. Engl J Med..

[27] Grothey A, Sargent DJ (2016). Adjuvant therapy for colon cancer: small steps toward precision medicine. JAMA Oncol.

[28] Allen JE, El-Deiry WS (2011). Oxaliplatin uses JNK to restore TRAIL sensitivity in cancer cells through Bcl-xL inactivation. Gastroenterology.

[29] Montazami N, Andish Kheir, Majidi M, Yousefi J, Yousefi M, Mohamadnejad B (2015). L. et al. siRNA-mediated silencing of MDR1 reverses the resistance to oxaliplatin in SW480/OxR colon cancer cells. Cell Mol Biol (Noisy-le-Gd).

[30] El Khoury F, Corcos L, Durand S, Simon B, Le Jossic-Corcos C (2016). Acquisition of anticancer drug resistance is partially associated with cancer stemness in human colon cancer cells. Int J Oncol..

[31] Chian S, Li YY, Wang XJ, Tang XW (2014). Luteolin sensitizes two oxaliplatin-resistant colorectal cancer cell lines to chemotherapeutic drugs via inhibition of the Nrf2 pathway. Asian Pac J Cancer Prev..

[32] Takahashi K, Tanaka M, Yashiro M, Matsumoto M, Ohtsuka A, Nakayama KI (2016). Protection of stromal cell-derived factor 2 by heat shock protein 72 prevents oxaliplatin-induced cell death in oxaliplatin-resistant human gastric cancer cells. Cancer Lett..

[33] Sui H, Zhou S, Wang Y, Liu X, Zhou L, Yin P (2011). COX-2 contributes to P-glycoprotein-mediated multidrug resistance via phosphorylation of c-Jun at Ser63/73 in colorectal cancer. Carcinogenesis.

[34] C.J. Massey Dawkins M.A., M.D., D.A., F.F.A.R.C.S. Br Med J. **3**, 493 (1975). 1098728

[35] Ma Y, Yang Y, Wang F, Moyer MP, Wei Q, Zhang P (2016). Long non-coding RNA CCAL regulates colorectal cancer progression by activating Wnt/β-catenin signalling pathway via suppression of activator protein 2α. Gut.

[36] Nagaraju GP, Wu C, Merchant N, Chen Z, Lesinski GB, El-Rayes BF (2017). Epigenetic effects of inhibition of heat shock protein 90 (HSP90) in human pancreatic and colon cancer. Cancer Lett.

[37] Xu R, Rai A, Chen M, Suwakulsiri W, Greening DW, Simpson RJ (2018). Extracellular vesicles in cancer - implications for future improvements in cancer care. Nat Rev Clin Oncol.

[38] Sousa D, Lima RT, Vasconcelos MH (2015). Intercellular Transfer of Cancer Drug Resistance Traits by Extracellular Vesicles. Trends Mol Med.

[39] Hu YB, Yan C, Mu L, Mi YL, Zhao H, Hu H (2019). Correction: Exosomal Wnt-induced dedifferentiation of colorectal cancer cells contributes to chemotherapy resistance. Oncogene.

[40] Liu T, Zhang X, Du L, Wang Y, Liu X, Tian H (2020). Correction to: Exosome-transmitted miR-128-3p increase chemosensitivity of oxaliplatin-resistant colorectal cancer. Mol Cancer.

