# Supplementary material for: DNAJB8 in small extracellular vesicles promotes Oxaliplatin resistance through TP53/MDR1 pathway in colon cancer
Source: Cell Death Dis. 2022 Feb 14;13(2):151. doi: 10.1038/s41419-022-04599-x (PMC8844036; doi:10.1038/s41419-022-04599-x)
Supplement: Supplementary file 2 — supplementary files [file 41419_2022_4599_MOESM2_ESM.docx]

**Supplementary Tables**

Table S1 Primers and probes used in the study

| Gene | Sequence | Product length  （bp） |
| --- | --- | --- |
| GAPDH | 5’- GAAAGCCTGCCGGTGACTAA -3’ | 150 |
|  | 3’- GCCCAATACGACCAAATCAGAG -5’ |  |
| TP53 | 5’- TGAAGCTCCCAGAATGCCAG -3’ | 107 |
|  | 3’- CAGAAGATGACAGGGGCCAG -5’ |  |
| DNAJB1 | 5’- CTTCCCCGAAAGGATTCCCC -3’ | 83 |
|  | 3’- AGTCCTTGGGGAGCTCAGAT -5’ |  |
| DNAJB2 | 5’- ACGACTGACCAGTTGCCAT -3’ | 91 |
|  | 3’- CGCCGATACGCCTTCTTGA -5’ |  |
| DNAJB3 | 5’- GTTGTCGGACGCCAAGAAAC -5’ | 145 |
|  | 3’- GAACTCCCTGAAGACGTCGG -3’ |  |
| DNAJB4 | 5’-TGTCATCACAGGACTAGGGAA -3’ | 135 |
|  | 3’-GAGGGCTTGTTTTCGGTAAGC -5’ |  |
| DNAJB5 | 5’-TCCACCAAGCGCATGAAGAT -3’ | 165 |
|  | 3’-AGCAGGGATGTTGTCAGGTG -5’ |  |
| DNAJB6 | 5’-ATGAAGTGCTGTCGGATGCT | 87 |
|  | 3’-GACTTCCACCTCCTCCTCCA |  |
| DNAJB7 | 5’-TGAAGACTCGCTTGAGGACC -3’ | 171 |
|  | 3’-GTAAGGCCTTCATGCCCCAA -5’ |  |
| DNAJB8 | 5’-CCATGGAAGAGGAACAAGCCT -3’ | 159 |
|  | 3’-CACATGTGGCTTAGCAGAGGA -5’ |  |
| DNAJB9 | 5’-GAGCGCCAAATCAAGAAGGC -3’ | 79 |
|  | 3’-CTTCAGCATCCGGGCTCTTA -5 |  |
| DNAJB10 | 5’-GCGTACCGAAAGAAGGCTCT -3’ | 188 |
|  | 3’-TCCGATCGAGAAGGACCACT -5’ |  |
| DNAJB11 | 5’-CTGCTATACCTCATCGGGGC -3’ | 146 |
|  | 3’-GGATCATCAGGGTTCCGGTC -5’ |  |
| DNAJB12 | 5’-CACCCACAGACACAACCCAT -3’ | 129 |
|  | 3’-CCCTTTTCACAGCTGCAACC -5’ |  |
| DNAJB13 | 5’-AGATCAAGCAGGCGTACCG -3’ | 126 |
|  | 3’-CTGGTTTCCGGTCACTCAGC -5’ |  |
| DNAJB14 | 5’-TGAAAAATGGAAGCACGGCTG -3’ | 168 |
|  | 3’-TACATTTGTTTATGCTGAGAACTCC -5’ |  |

Table S2 DNAJB8 expression in colorectal cancer

| Clinical or molecular feature | Total *N* | DNAJB8 | | |
| --- | --- | --- | --- | --- |
|  |  | High | Low | *P* |
|  | 216 | 93 | 123 |  |
| Age (mean±SD) |  |  |  | *0.216* |
| ＜60 | 107 | 51 | 56 |  |
| ≥60 | 109 | 42 | 67 |  |
| Gender |  |  |  | *0.785* |
| Male | 109 | 48 | 61 |  |
| Female | 107 | 45 | 62 |  |
| Location |  |  |  | *0.450* |
| Colon | 63 | 30 | 33 |  |
| Rectum | 153 | 63 | 90 |  |
| Grade |  |  |  | *0.591* |
| Low | 178 | 75 | 103 |  |
| High | 38 | 18 | 20 |  |
| Stage |  |  |  | *0.011* |
| Ⅱ | 8 | 0 | 8 |  |
| Ⅲ | 208 | 93 | 115 |  |
| Large |  |  |  | *0.584* |
| ≤ 14cm^3^ | 111 | 50 | 61 |  |
| > 14cm^3^ | 105 | 43 | 62 |  |
| T |  |  |  | *0.867* |
| T3 | 170 | 74 | 96 |  |
| T4 | 46 | 19 | 27 |  |
| N |  |  |  | *0.011* |
| N0 | 8 | 0 | 8 |  |
| N1+N2 | 208 | 93 | 115 |  |

Table S3 Univariate and multivariate analysis for Relapse-free survival (Cox proportional hazards regression model)

| Characteristics | Univariate analysis | | |  | Multivariate analysis | | |
| --- | --- | --- | --- | --- | --- | --- | --- |
|  | HR | 95% CI | *P* |  | HR | 95% CI | *P* |
| Gender (male vs female) | 0.815 | 0.540-1.229 | *0.329* |  | 0.813 | 0.532-1.243 | *0.339* |
| Age (≤ 60 years vs > 60 years) | 1.207 | 0.801-1.818 | *0.368* |  | 1.183 | 0.772-1.813 | *0.440* |
| Location (Colon vs Rectum) | 1.876 | 1.233-2.854 | *0.003* |  | 1.414 | 0.870-2.296 | *0.162* |
| Grade (low vs high) | 3.023 | 1.936-4.722 | *0.000* |  | 3.404 | 2.127-5.447 | *0.000* |
| Large (≤ 14cm^3^ vs > 14cm^3^) | 1.108 | 0.736-1.668 | *0.623* |  | 0.815 | 0.527-1.262 | *0.359* |
| Stage (phage) | 2.089 | 1.338-3.259 | *0.001* |  | — | — | *—* |
| pT (T3 vs T4) | 2.089 | 1.338-3.259 | *0.001* |  | 2.102 | 1.230-3.592 | *0.007* |
| pN (N0 vs N1+N2) | 1.045 | 0.331-3.301 | *0.941* |  | 1.472 | 0.431-5.027 | *0.537* |
| DNAJB8 (high vs low expression) | 1.981 | 1.313-2.990 | *0.001* |  | 1.940 | 1.272-2.961 | *0.002* |

**Supplementary Figures**


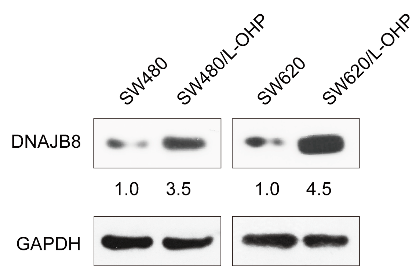


**Figure. S1 Expression of P-gP is analyzed by western blot assay in parental cells compared with L-OHP-resistant COAD cells.**

**
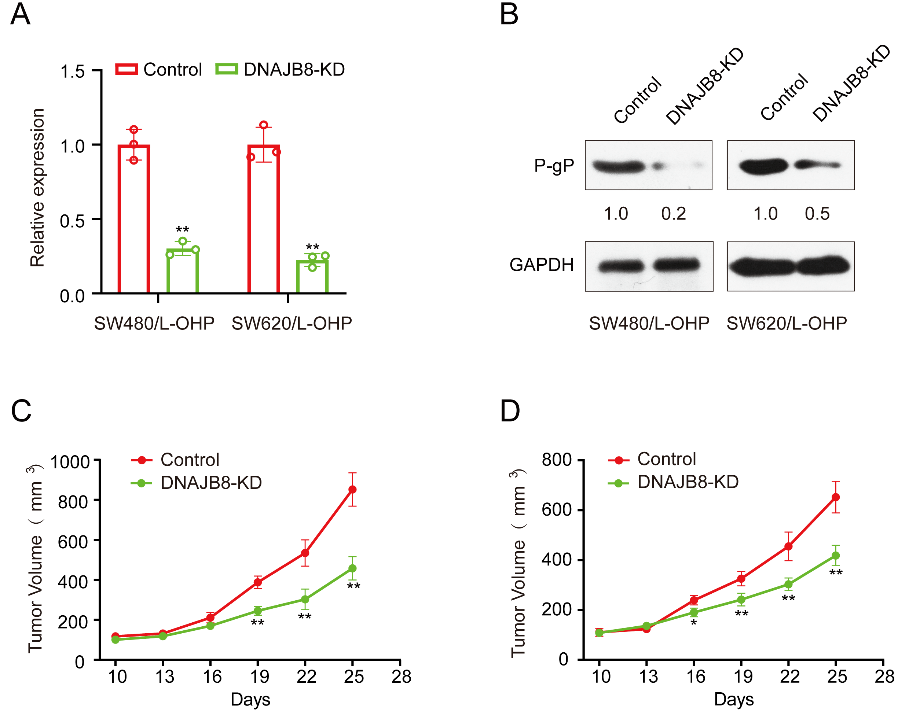
**

**Figure. S2 DNAJB8 silencing promotes COAD cells to L-OHP chemosensitivity.** Expression of MDR1(P-gP) was analyzed by qRT-PCR (A) and western blot assay (B) in L-OHP-resistant COAD cells transfected with DNAJB8 specific siRNA. (C) Subcutaneous xenograft assay of DNAJB8 knockdown SW620/L-OHP and SW480/L-OHP cells in nude mice with L-OHP treatment. Volumes of tumors are shown (n = 5 per group). Results shown are mean ± s.d. from a representative experiment. *p < 0.05; **p < 0.01; Student’s t test. Similar results were obtained in three independent experiments.


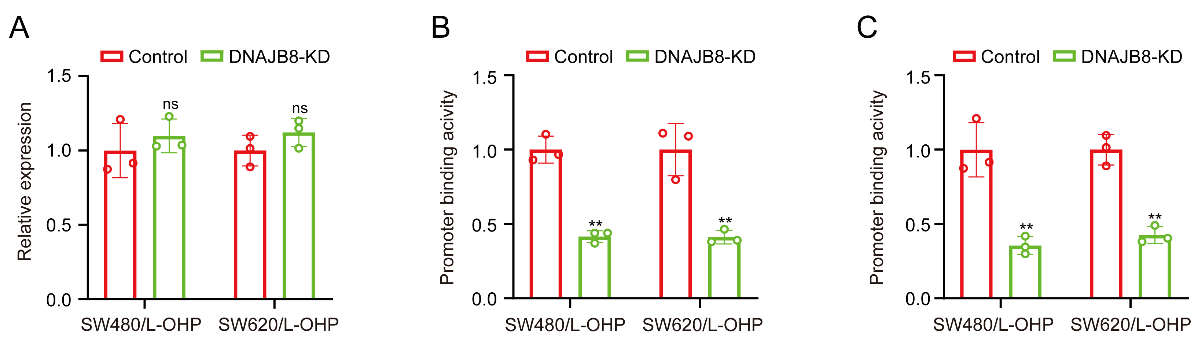


**Figure. S3 DNAJB8 knockdown inhibits the DNA binding ability of TP53 in L-OHP resistant COAD cells.** (A) The mRNA expression of TP53 was evaluated using qTR-PCR in L-OHP resistant COAD cells following DNAJB8 knockdown. (B) ID2 and (C) CXCL5 promoter binding activity by TP53 was detected in L-OHP-resistant colon cancer cells using a luciferase reporter gene assay. Results shown are mean ± s.d. from a representative experiment. *p < 0.05; **p < 0.01; Student’s t test. Similar results were obtained in three independent experiments.


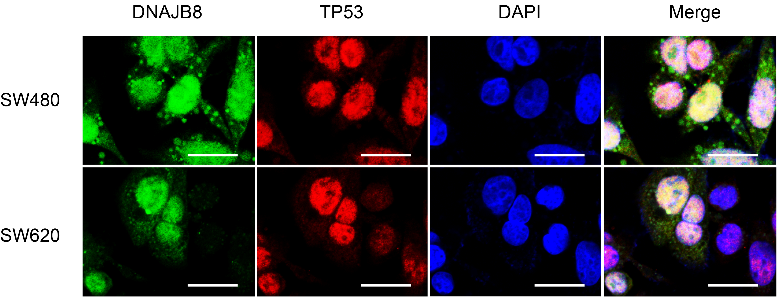


**Figure. S4 The co-localization of DNAJB8 and TP53 is evaluated in L-OHP resistant cancer cells using confocal microscopy.** Scale bar = 100µm.


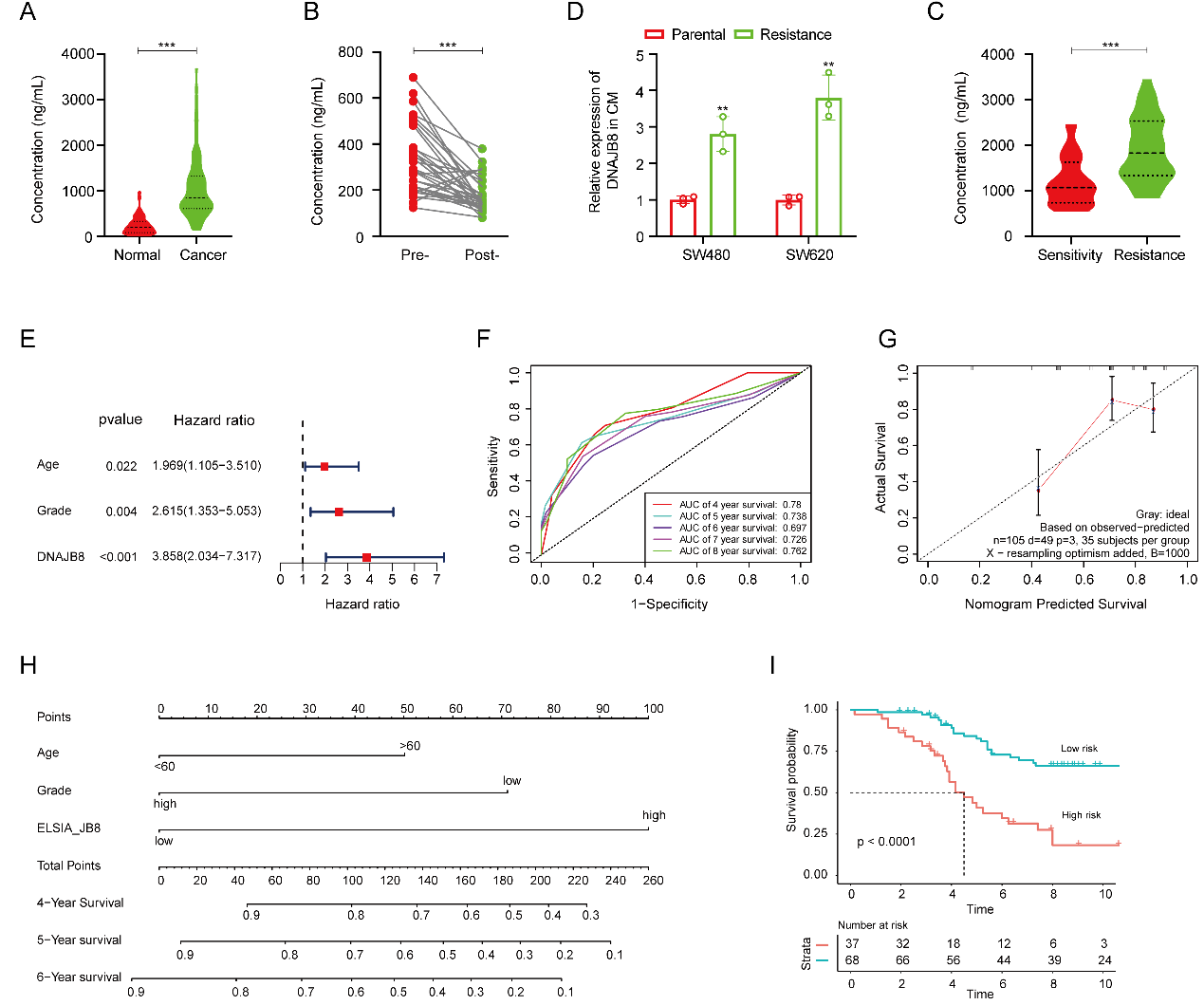


**Figure. S5 DNAJB8 Levels in serum correlate with L-OHP response in COAD patients.** (A) The serum DNAJB8 levels were evaluated using ELISA in COAD patients and healthy donors. (B) ELISA analysis of DNAJB8 in the serum of COAD patients before and after tumor resection. (C) ELISA analysis of DNAJB8 in the CM of L-OHP-resistant and parental COAD cells. (D) ELISA analysis of DNAJB8 in the serum of L-OHP resistant and sensitivity COAD patients. (E) The results of multivariate COX regression analysis. (F) The calibration curve for predicting patient survival at 5 years, the nomogram-predicted probability of overall survival is plotted on the x-axis; the actual overall survival is plotted on the y-axis. (G) The results of receiver operating characteristic (ROC). (H) Prognostic nomogram for COAD. (I) Kaplan–Meier curve analyses. Results shown are mean ± s.d. from a representative experiment. *p < 0.05; **p < 0.01; Student’s t test. Similar results were obtained in three independent experiments.


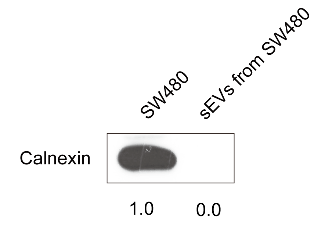


**Figure. S6 Calnexin expression was detected by western blot assay in SW480 cell and sEV from SW480 cells.**

**
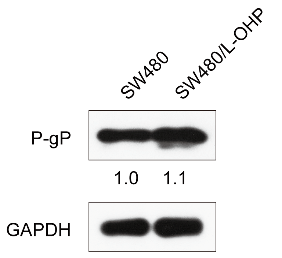
**

**Figure. S7 P-gP expression is detected by western blot assay in sEVs derived from resistant cells and parental cells.**


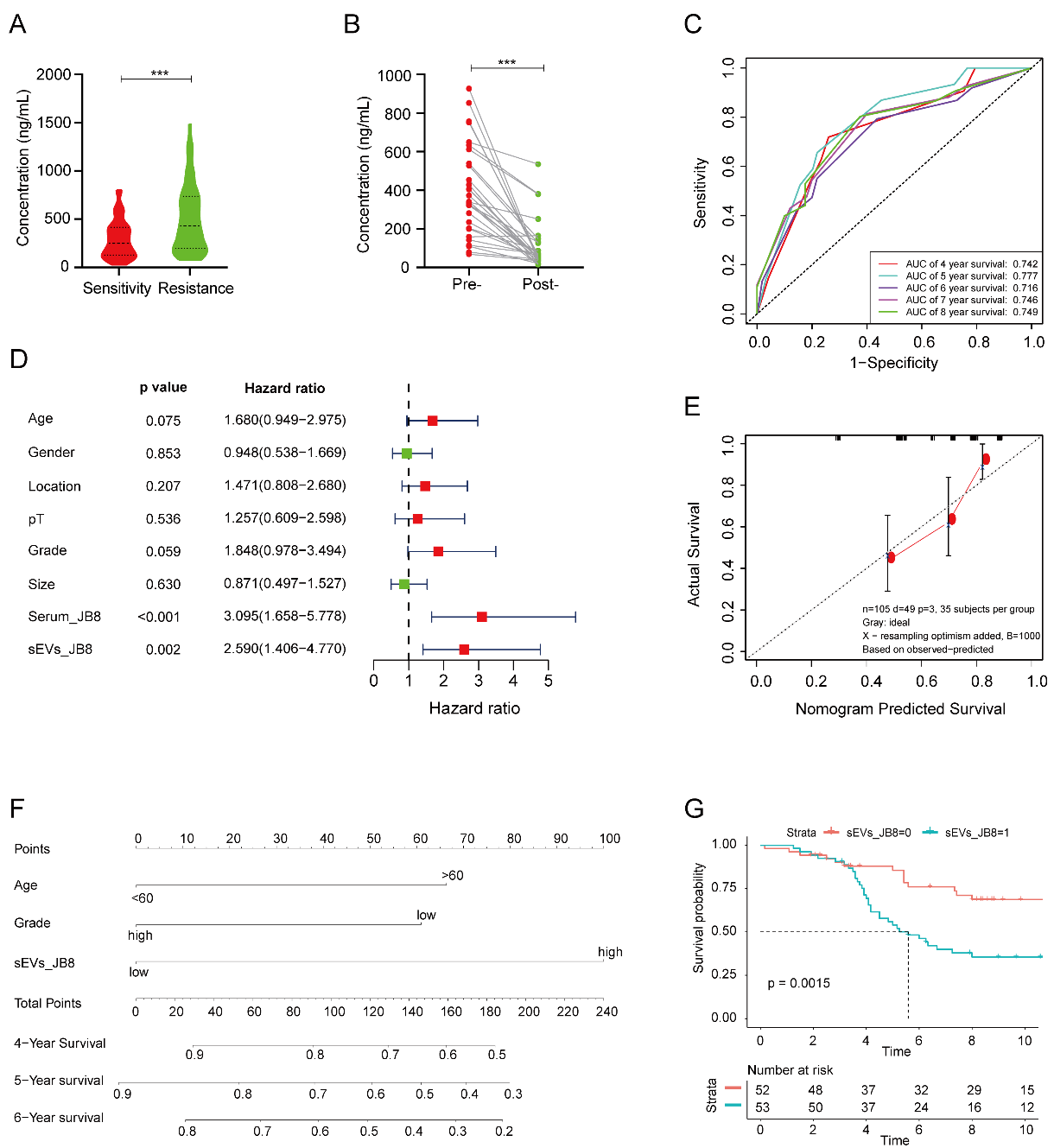


**Figure. S8 DNAJB8 levels in sEVs from COAD patient serum correlate with L-OHP response.** (A) The DNAJB8 from mediated serum sEVs was evaluated using ELISA in COAD patients and healthy donors. (B) The DNAJB8 from mediated serum sEVs was evaluated using ELISA in the serum of COAD patients before and after tumor resection. (C) The results of multivariate COX regression analysis. (D) The calibration curve for predicting patient survival at 5 years, the nomogram-predicted probability of overall survival is plotted on the x-axis, and the actual overall survival is plotted on the y-axis. (E) The results of the receiver operating characteristic (ROC). (F) Prognostic nomogram for COAD. (G) Kaplan–Meier curve analyses. Results shown are mean ± s.d. from a representative experiment. *p < 0.05; **p < 0.01; Student’s t test. Similar results were obtained in three independent experiments.


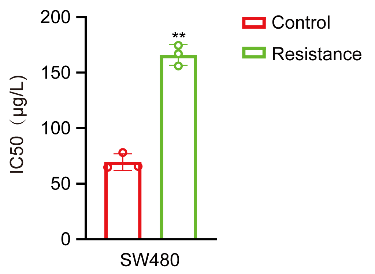


**Figure. S9 IC_50_ values of 5-FU in SW480 cells treated with 5-FU using CCK-8 assay.** Results shown are mean ± s.d. from a representative experiment. *p < 0.05; **p < 0.01; Student’s t test. Similar results were obtained in three independent experiments.


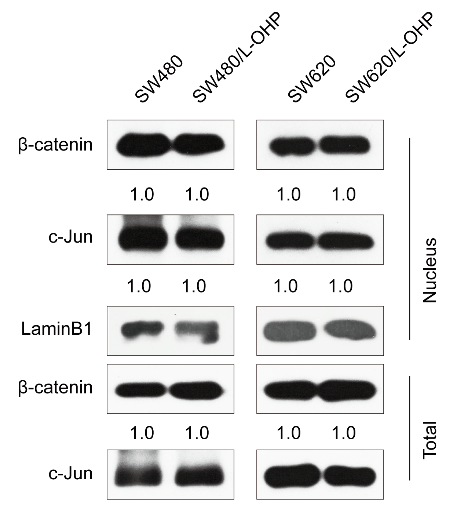


**Figure. S10 Nuclear translocation c-Jun and β-catenin is evaluated by western blot assay between L-OHP resistant cells and parental cells.**


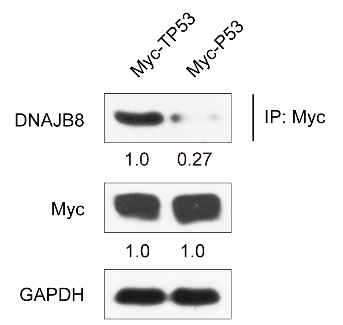


**Figure. S11 The interaction between wild-type P53 and DNAJB8 is detected using the co-IP assay in 293T cells.**

**
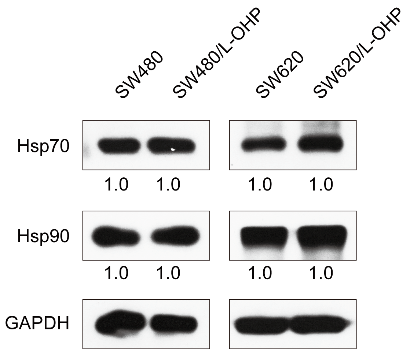
**

**Figure. S12 Hsp70 and Hsp90 expression is analyzed in the L-OHP resistant COAD cells and parental cells by western blot assay.**

**Supplementary codes**

#forest plot

rt <- read.table("mul.txt",header=T,sep="\t",row.names=1,check.names=F)

gene <- rownames(rt)

hr <- sprintf("%.3f",rt$"HR")

hrLow <- sprintf("%.3f",rt$"HR.95L")

hrHigh <- sprintf("%.3f",rt$"HR.95H")

Hazard.ratio <- paste0(hr,"(",hrLow,"-",hrHigh,")")

pVal <- ifelse(rt$pvalue<0.001, "<0.001", sprintf("%.3f", rt$pvalue))

pdf(file="forest-unicox.pdf", width = 10,height =12)

n <- nrow(rt)

nRow <- n+1

ylim <- c(1,nRow)

layout(matrix(c(1,2),nc=2),width=c(3,2))

xlim = c(0,3)

par(mar=c(4,2.5,2,1))

plot(1,xlim=xlim,ylim=ylim,type="n",axes=F,xlab="",ylab="")

text.cex=0.8

text(0,n:1,gene,adj=0,cex=text.cex)

text(1.5-0.5*0.2,n:1,pVal,adj=1,cex=text.cex);text(1.5-0.5*0.2,n+1,'pvalue',cex=text.cex,font=2,adj=1)

text(3,n:1,Hazard.ratio,adj=1,cex=text.cex);text(3,n+1,'Hazard ratio',cex=text.cex,font=2,adj=1,)

par(mar=c(4,1,2,1),mgp=c(2,0.5,0))

xlim = c(0,max(as.numeric(hrLow),as.numeric(hrHigh)))

plot(1,xlim=xlim,ylim=ylim,type="n",axes=F,ylab="",xaxs="i",xlab="Hazard ratio")

arrows(as.numeric(hrLow),n:1,as.numeric(hrHigh),n:1,angle=90,code=3,length=0.05,col="darkblue",lwd=2.5)

abline(v=1,col="black",lty=2,lwd=2)

boxcolor = ifelse(as.numeric(hr) > 1, 'red', 'green')

points(as.numeric(hr), n:1, pch = 15, col = boxcolor, cex=1.3)

axis(1)

dev.off()

#DCA

library(rms)

library(rmda)

non_tumor<-read.table("clinical.txt",header=T,sep="\t")

modul1<- decision_curve(status~

,data= non_tumor,

family = binomial(link ='logit'),

thresholds= seq(0,1, by = 0.01),

confidence.intervals = 0.95)

modul2<- decision_curve(status~

,data= non_tumor,

family = binomial(link ='logit'),

thresholds= seq(0,1, by = 0.01),

confidence.intervals = 0.95)

modul3<- decision_curve(status~

,data= non_tumor,

family = binomial(link ='logit'),

thresholds= seq(0,1, by = 0.01),

confidence.intervals = 0.95)

pdf("DCA.pdf")

plot_decision_curve(list(modul1,modul2,modul3),

curve.names= c("","", ""), xlab="Threshold probability",

cost.benefit.axis =FALSE,col=c( "blue","red","purple"),

confidence.intervals=FALSE,

standardize = FALSE)

dev.off()

#ROC

library(survival)

library(timeROC)

lncRNA<-read.table("risk_score.txt",header=T,sep="\t")

predict_1_year<- 1

predict_2_year<- 2

predict_3_year<- 3

predict_4_year<- 4

predict_5_year<- 5

ROC<-timeROC(T=lncRNA$OS,delta=lncRNA$vital_status,

marker=lncRNA$risk_score,cause=1,

weighting="marginal", times=c(predict_1_year,predict_2_year,predict_3_year,predict_4_year,predict_5_year),ROC=TRUE)

pdf("ROC11.pdf")

plot(ROC,time=predict_1_year,title=FALSE,lwd=3)

plot(ROC,time=predict_2_year,col="turquoise",add=TRUE,title=FALSE,lwd=3)

plot(ROC,time=predict_3_year,col="purple",add=TRUE,title=FALSE,lwd=3)

plot(ROC,time=predict_4_year,col="magenta",add=TRUE,title=FALSE,lwd=3)

plot(ROC,time=predict_5_year,col="green",add=TRUE,title=FALSE,lwd=3)

legend("bottomright",

c(paste("AUC of 1 year survival: ",round(ROC$AUC[1],3)),

paste("AUC of 2 year survival: ",round(ROC$AUC[2],3)),

paste("AUC of 3 year survival: ",round(ROC$AUC[3],3)),

paste("AUC of 4 year survival: ",round(ROC$AUC[4],3)),

paste("AUC of 5 year survival: ",round(ROC$AUC[5],3))),col=c("red","turquoise","purple","magenta","green"),lwd=2)

dev.off()

#Univariable COX

library(survival)

library(rms)

library(foreign)

tcga<-read.table(".txt",header=T,sep="\t")

tcga$age<-factor(tcga$age,labels=c())

tcga$sex<-factor(tcga$sex,labels=c())

tcga$histological_type<-factor(tcga$histological_type,labels=c())

tcga$T<-factor(tcga$T,labels=c())

tcga$N<-factor(tcga$N,labels=c())

tcga$M<-factor(tcga$M,labels=c())

tcga$Stage<-factor(tcga$Stage,labels=c())

tcga$Grade<-factor(tcga$Grade,labels=c())

tcga$score<-factor(tcga$score,labels=c())

ddist <- datadist(tcga)

options(datadist='ddist')

fmla1 <- as.formula(Surv(OS,vital_status) ~)

cox2 <- coxph(fmla1,data=tcga)

summary(cox2)

#Mulvariable COX

library(survival)

library(rms)

library(foreign)

tcga<-read.table("clinical1.txt",header=T,sep="\t")

tcga$stage<-factor(tcga$stage,labels=c())

tcga$score<-factor(tcga$score,labels=c())

ddist <- datadist(tcga)

options(datadist='ddist')

fmla1 <- as.formula(Surv(0S,Vital_status) ~)

cox2 <- coxph(fmla1,data=tcga)

summary(cox2)

library(survminer)

pdf("forest.pdf")

ggforest(cox2,fontsize = 0.7)

dev.off()

cox <- cph(Surv(OS,Vital_Status) ~,surv=T,x=T, y=T,data=tcga)

surv <- Survival(cox)

surv <- Survival(cox)

sur_3_year<-function(x)surv(1*365*3,lp=x)

sur_5_year<-function(x)surv(1*365*5,lp=x)

sur_7_year<-function(x)surv(1*365*7,lp=x)

nom_sur <- nomogram(cox,fun=list(sur_3_year,sur_5_year,sur_7_year),lp= F,funlabel=c('3-Year Survival','5-Year survival','7-Year survival'),maxscale=100,fun.at=c('0.9','0.8','0.7','0.6','0.5','0.4','0.3','0.2','0.1'))

pdf("nom.pdf",15,10)

plot(nom_sur,xfrac=0.25)

dev.off()

cox1 <- cph(Surv(OS,Vital_Status) ~,surv=T,x=T, y=T,time.inc = 1*365*3,data=tcga)

cal <- calibrate(cox1, cmethod="KM", method="boot", u=1*365*3, m=142 , B=1000)

pdf("calibrate-3.pdf",8,6)

par(mar = c(10,5,3,2),cex = 1.0)

plot(cal,lwd=2,lty=1,errbar.col="black",xlim = c(0,1),ylim = c(0,1),xlab ="Nomogram Predicted Survival",ylab="Actual Survival",col="red")

lines(cal,c('mean.predicted',‘KM'),type = ‘a',lwd = 2,col ="black" ,pch = 16)

mtext(“ ”)

box(lwd = 1)

abline(0,1,lty = 3,lwd = 2,col = "black")

dev.off()

cox2 <- coxph(Surv(RFS,vital_status) ~stage + risk_level,data=tcga)

risk_score<-predict(cox2,type="risk",newdata=tcga)

risk_level<-as.vector(ifelse(risk_score>median(risk_score),"High","Low"))

write.table(cbind(id=rownames(cbind(tcga[,1:2],risk_score,risk_level)),cbind(tcga[,1:2],risk_score,risk_level)),"risk_score_all.txt",sep="\t",quote=F,row.names=F)

#KM

library(survival)

library(survminer)

inputdata<- read.table("KM1.txt",header=T,sep="\t")

fit <- survfit(Surv(OS,vital_status)~ group,data = inputdata)

ggsurvplot(

fit,

risk.table = TRUE,

pval = TRUE,

conf.int = FALSE,

surv.median.line="hv",

xlim = c(0,20),

break.time.by = 5,

risk.table.y.text.col = T,

risk.table.y.text = FALSE

)
