# Supplementary material for: DNAJB8 in small extracellular vesicles promotes Oxaliplatin resistance through TP53/MDR1 pathway in colon cancer
Source: Cell Death Dis. 2022 Feb 14;13(2):151. doi: 10.1038/s41419-022-04599-x (PMC8844036; doi:10.1038/s41419-022-04599-x)

Figure 2A

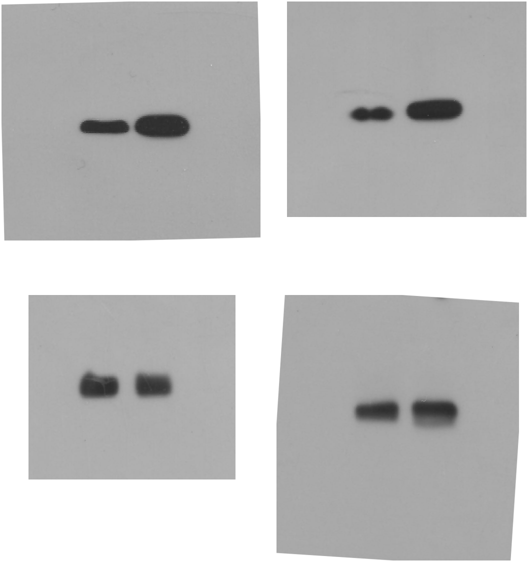

Figure 3D

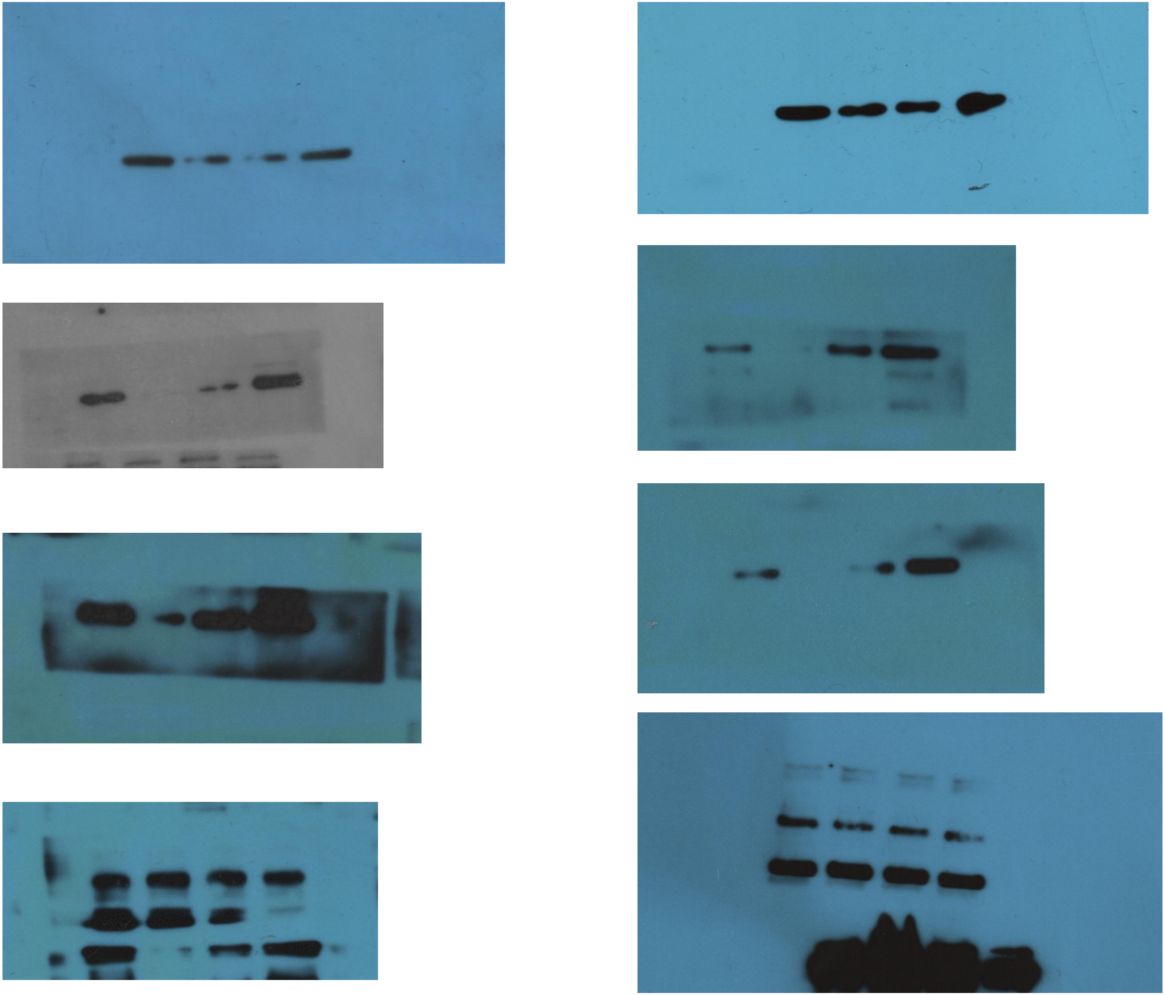

Figure 3E

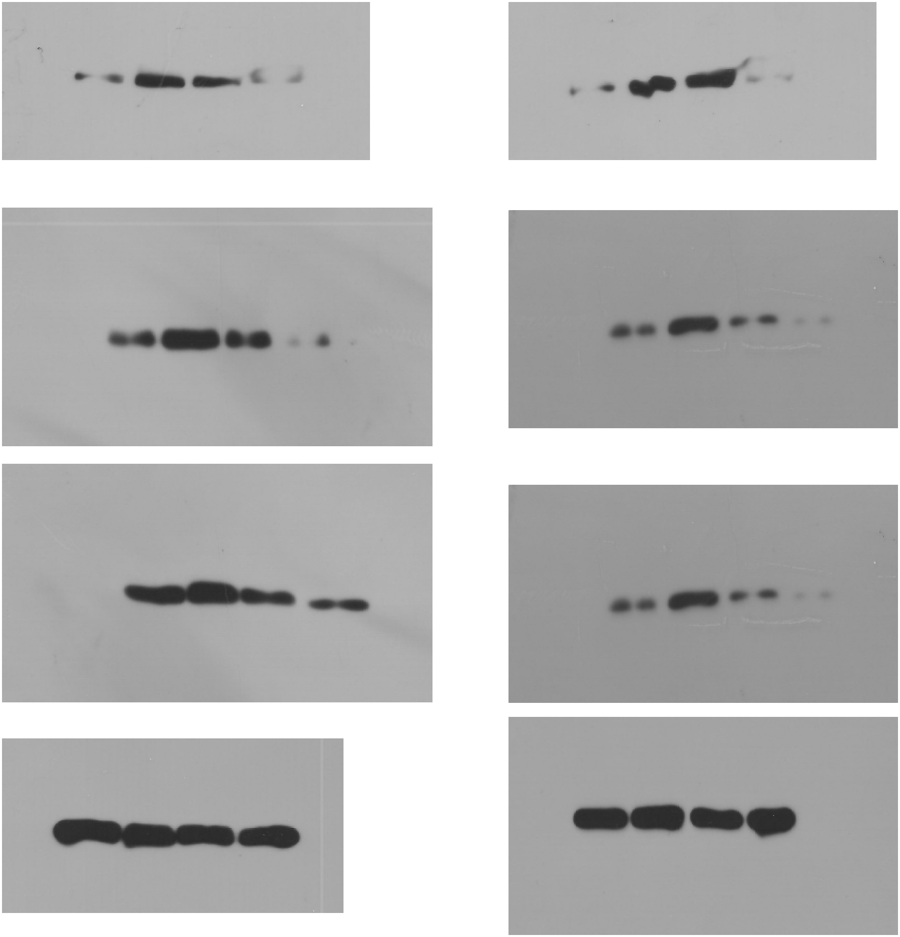

Figure 3F

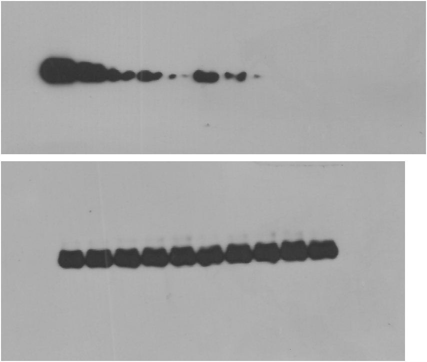

Figure 3G

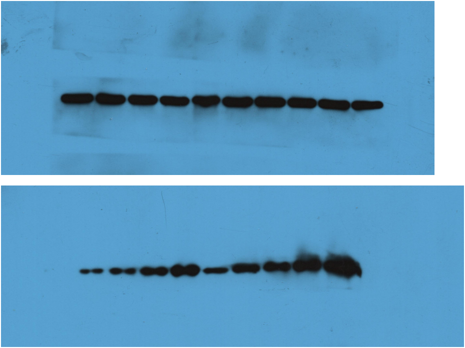

Figure 3H

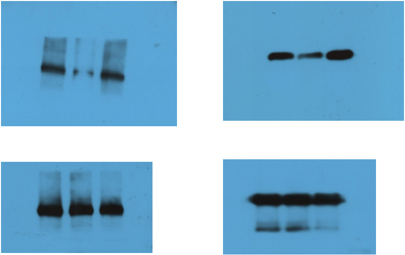

Figure 3I

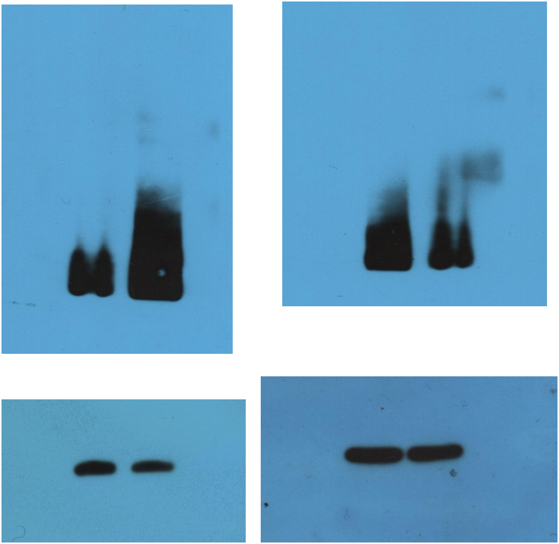

Figure 4A

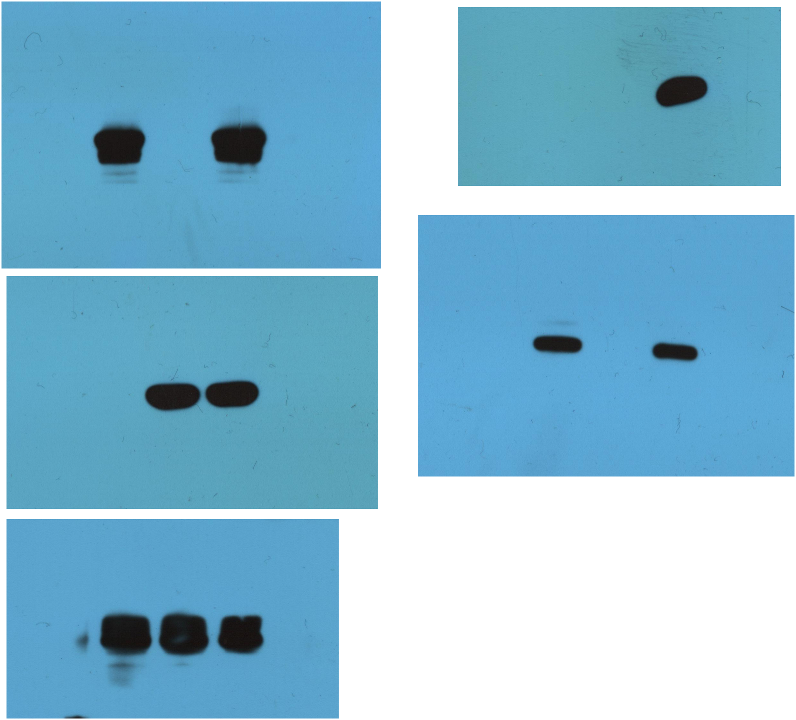

Figure 4B

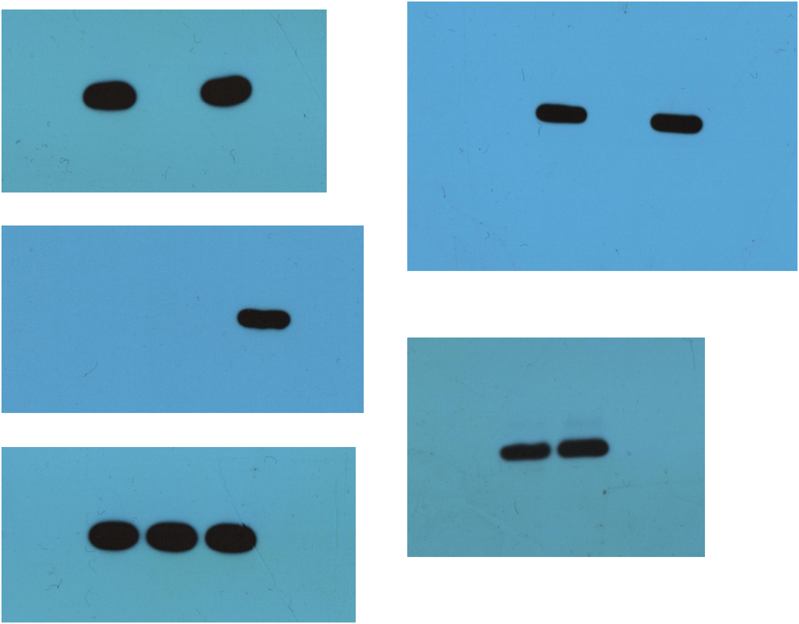

Figure 4C

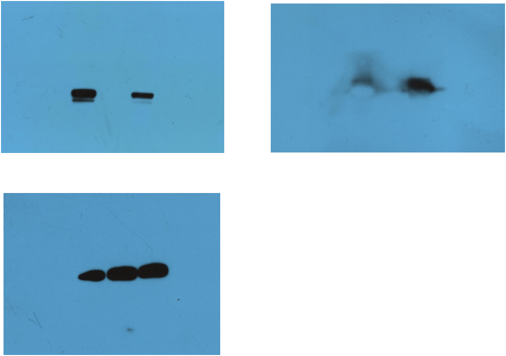

Figure 4D

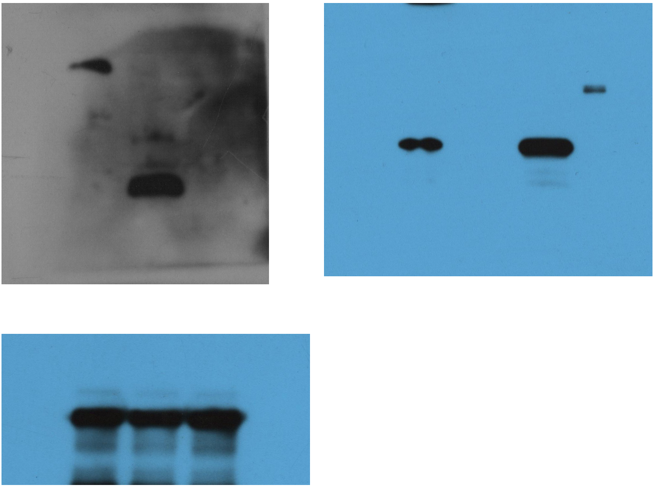

Figure 6E

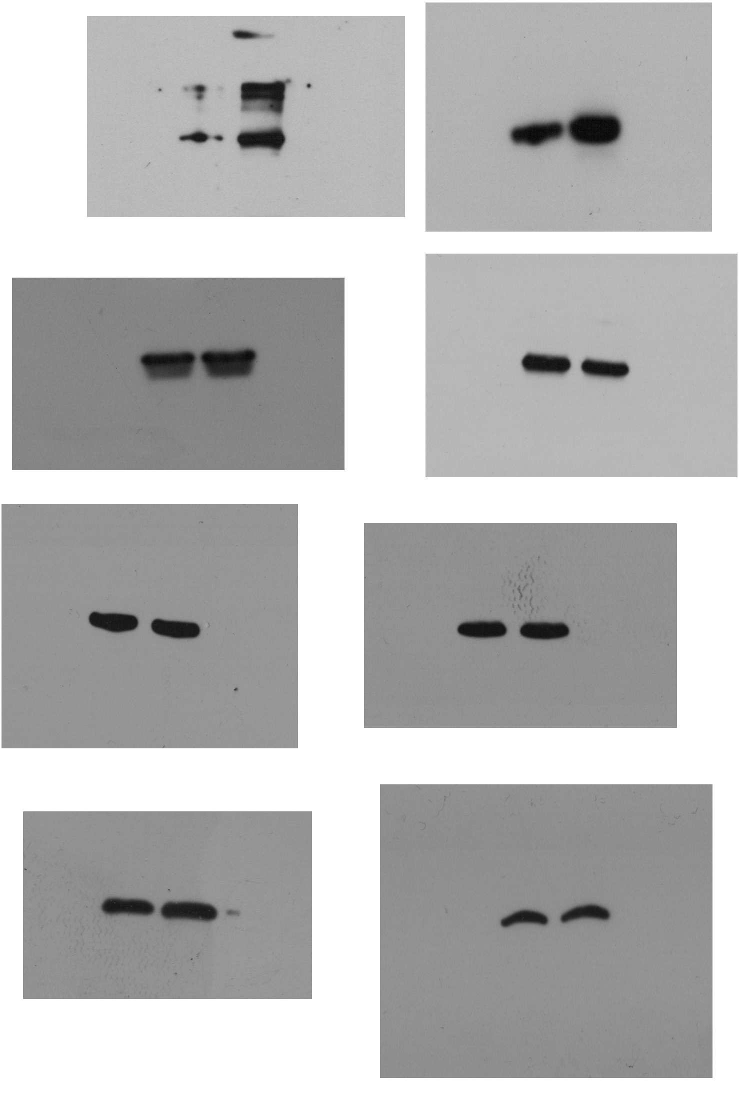

Figure 6G

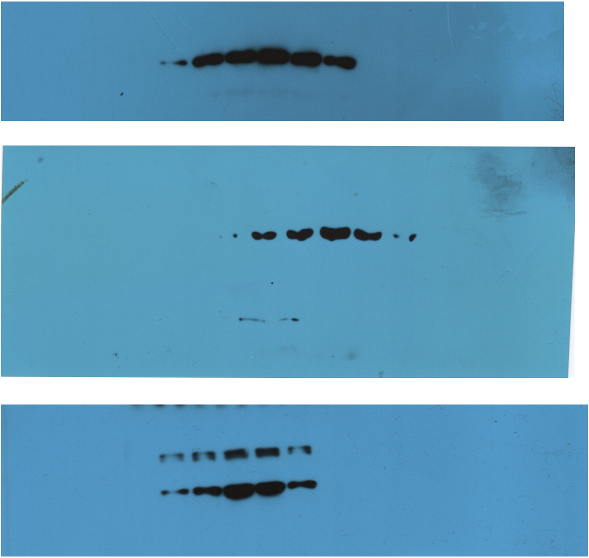

Figure 6I

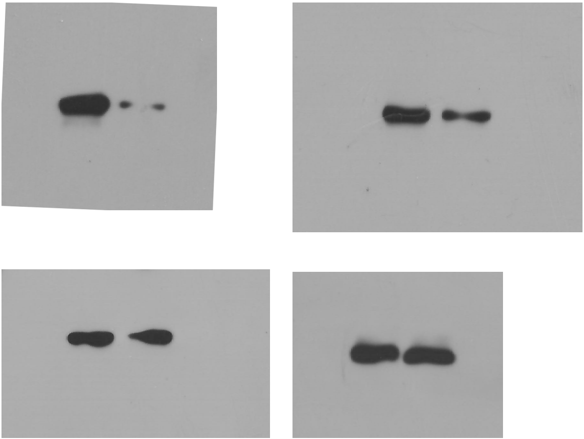

Figure 7A

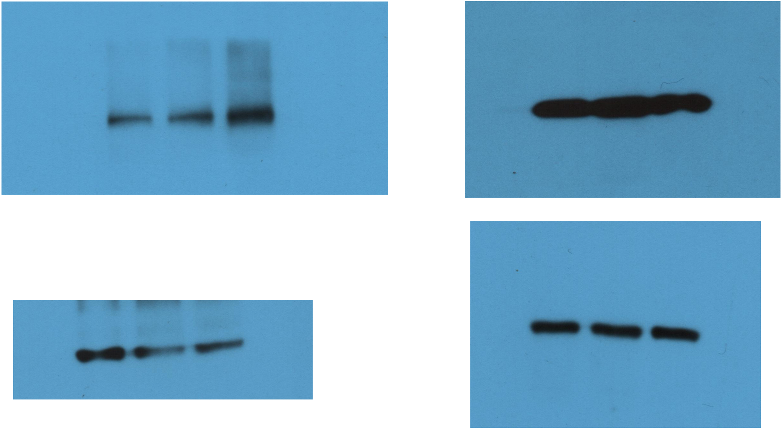

Figure 7B

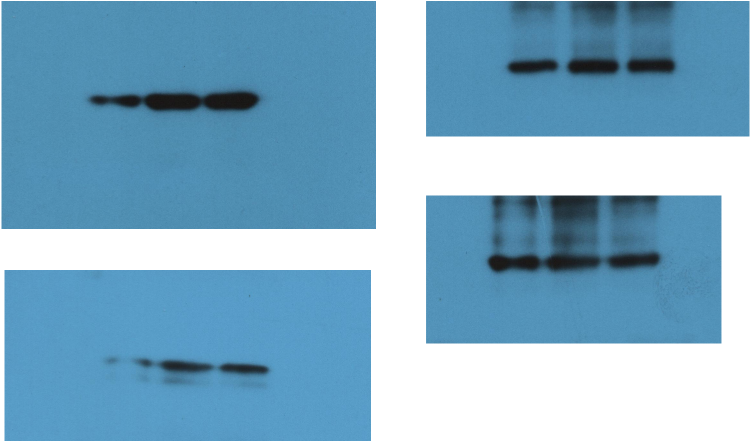

Figure 7H

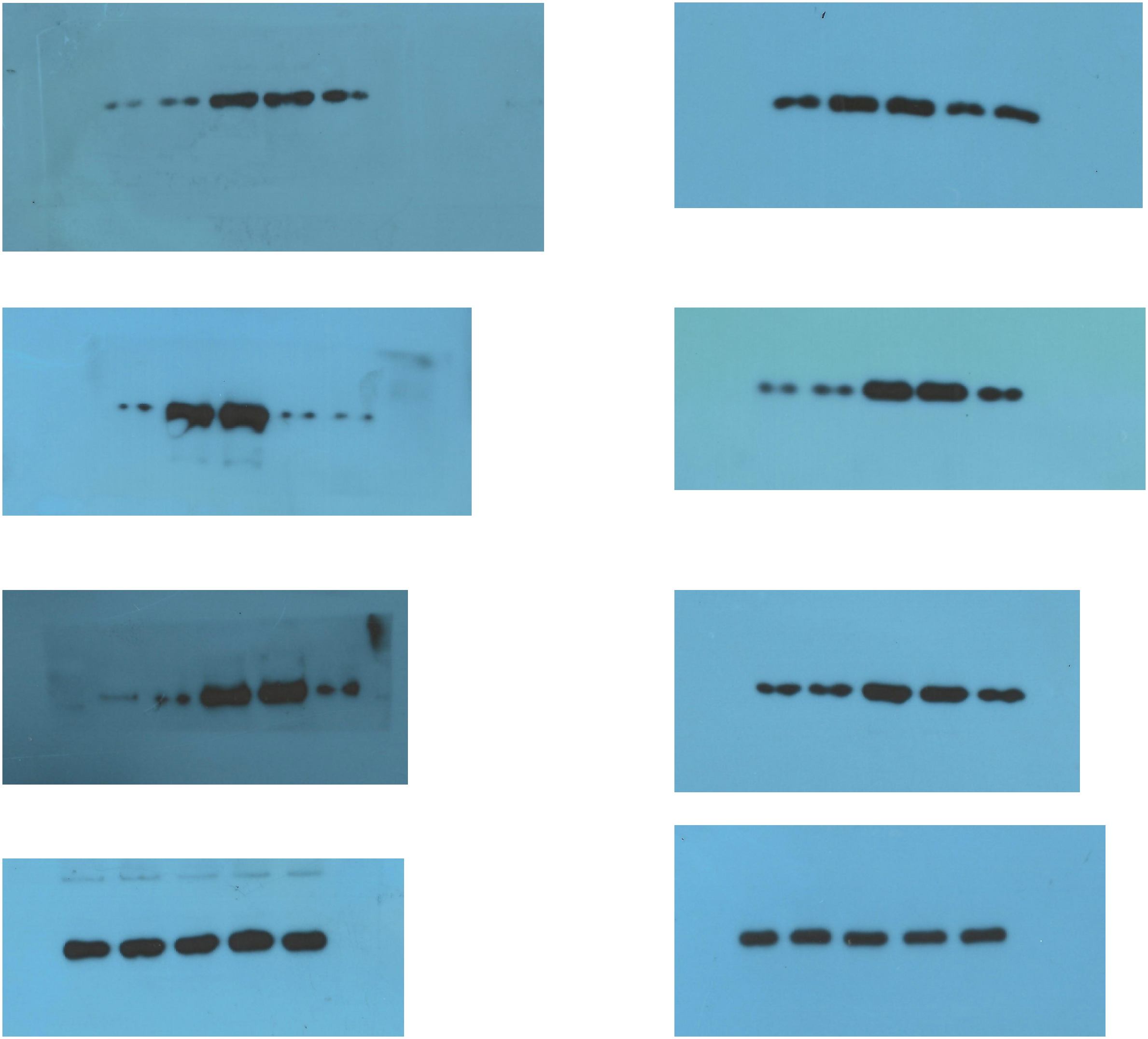

Figure S2

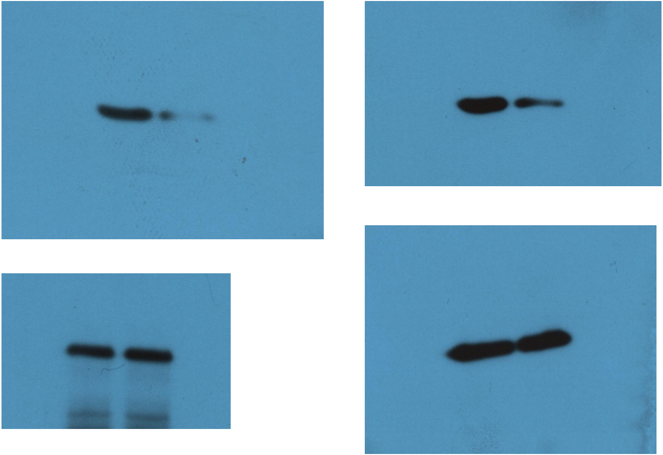

Figure S

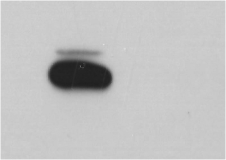

Figure S6

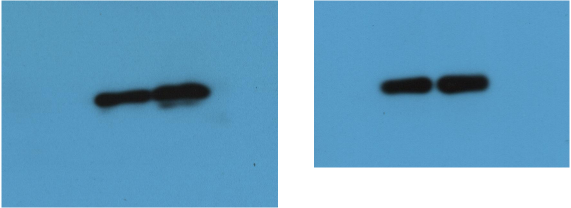

Figure S10

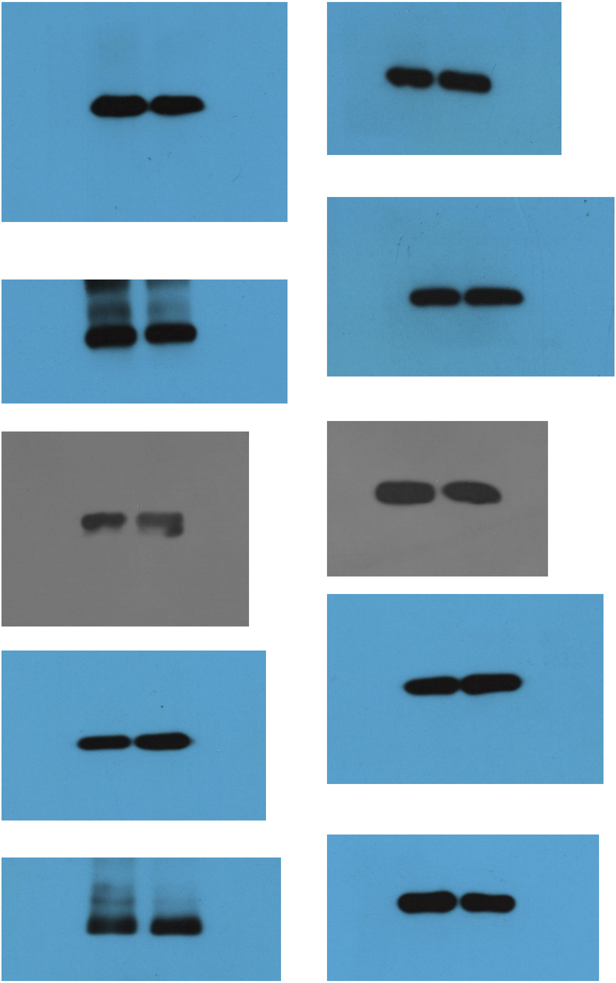

Figure S11

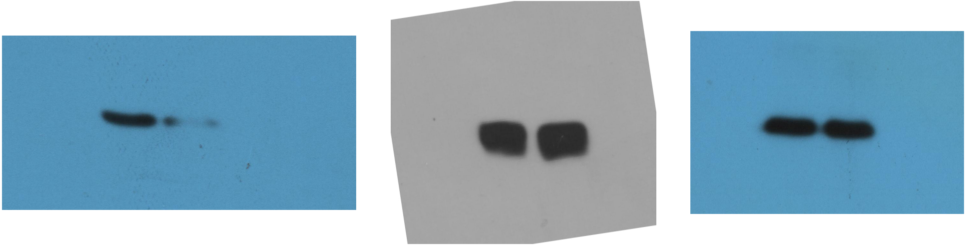

Figure S12

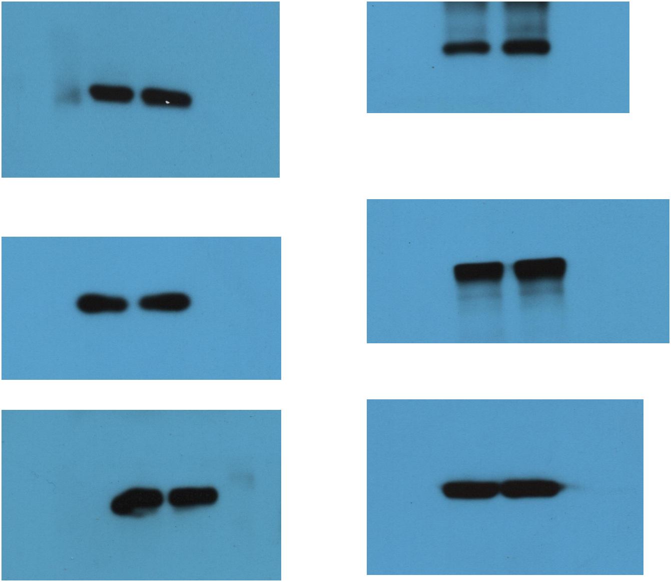

Supplement: Supplementary file 3 — Raw data of WB [file 41419_2022_4599_MOESM3_ESM.pdf]
